# Matrix stiffening promotes chondrocyte senescence and the osteoarthritis development through downregulating *HDAC3*

**DOI:** 10.1038/s41413-024-00333-9

**Published:** 2024-05-24

**Authors:** Bowen Fu, Jianlin Shen, Xuenong Zou, Nian Sun, Ze Zhang, Zengping Liu, Canjun Zeng, Huan Liu, Wenhua Huang

**Affiliations:** 1 Guangdong Medical Innovation Platform for Translation of 3D Printing Application, The Third Affiliated Hospital, Southern Medical University, Guangzhou, 510630 Guangdong China; 2 Guangdong Engineering Research Center for Translation of Medical 3D Printing Application, Guangdong Provincial Key Laboratory of Digital Medicine and Biomechanics, National Key Discipline of Human Anatomy, School of Basic Medical Sciences, Southern Medical University, Guangzhou, 510145 Guangdong China; 3 Department of Foot and Ankle Surgery, Center for Orthopedic Surgery, The Third Affiliated Hospital, Southern Medical University, Guangzhou, 510630 Guangdong China; 4 Department of Orthopedics, Affiliated Hospital of Putian University, Putian, 351100 Fujian China; 5 Central Laboratory, Affiliated Hospital of Putian University, Putian, 351100 Fujian China; 6 Guangdong Provincial Key Laboratory of Orthopedics and Traumatology, Department of Spinal Surgery, The First Affiliated Hospital of Sun Yat-sen University, Guangzhou, 510080 Guangdong China; 7 Nanfang Hospital, Southern Medical University, Guangzhou, 510515 Guangdong China; 8 Guangzhou Blood Center, Guangzhou, 510095 Guangdong China; 9 Department of Orthopedics, The Affiliated Traditional Chinese Medicine Hospital, Southwest Medical University, Luzhou, 646000 Sichuan China

**Keywords:** Bone quality and biomechanics, Bone

## Abstract

Extracellular matrix (ECM) stiffening is a typical characteristic of cartilage aging, which is a quintessential feature of knee osteoarthritis (KOA). However, little is known about how ECM stiffening affects chondrocytes and other molecules downstream. This study mimicked the physiological and pathological stiffness of human cartilage using polydimethylsiloxane (PDMS) substrates. It demonstrated that epigenetic Parkin regulation by histone deacetylase 3 (*HDAC3*) represents a new mechanosensitive mechanism by which the stiffness matrix affected chondrocyte physiology. We found that ECM stiffening accelerated cultured chondrocyte senescence in vitro, while the stiffness ECM downregulated HDAC3, prompting Parkin acetylation to activate excessive mitophagy and accelerating chondrocyte senescence and osteoarthritis (OA) in mice. Contrarily, intra-articular injection with an HDAC3-expressing adeno-associated virus restored the young phenotype of the aged chondrocytes stimulated by ECM stiffening and alleviated OA in mice. The findings indicated that changes in the mechanical ECM properties initiated pathogenic mechanotransduction signals, promoted the Parkin acetylation and hyperactivated mitophagy, and damaged chondrocyte health. These results may provide new insights into chondrocyte regulation by the mechanical properties of ECM, suggesting that the modification of the physical ECM properties may be a potential OA treatment strategy.

## Introduction

Osteoarthritis (OA) is the most common age-related and post-traumatic degenerative joint disease.^1^ There are more than 300 million patients with knee osteoarthritis (KOA) and is projected to become the disease with the highest disability rate globally by 2030.^2,3^ Although most of the etiological factors are still under investigation,^4^ advanced age and mechanical overloading recognized as the two most significant risk factors for OA development.^5^ All cells in the human body are subject to mechanical influences.^6^ This is particularly true of articular cartilage, given that its primary role is to transmit force to the underlying bone and decrease friction in the joint.^7,8^ Appropriate mechanical loading is essential for the health of articular cartilage, while mechanical overloading can result in degenerative lesions of articular cartilage, which can lead to the occurrence and development of OA.^9,10^

However, the specific mechanism behind the role of mechanical overloading in cartilage degeneration remains unclear. This is partially due to several factors. (1) Gaps in our understanding of whether the currently studied mechanical models of OA can summarize human disease. So far, most mechanical studies have used compression, tension, and shear force models for chondrocytes, while there is limited exploration on the stress of chondrocytes in patients with OA at daily resting state^11,12^; (2) The knowledge regarding the molecular mechanisms driving disease development is incomplete, particularly for KOA. KOA is characterized by interference between cells and the extracellular matrix (ECM) where they are located, leading to anabolic and catabolic imbalances. No clear inciting event is evident in most cases.

The primary constituents of cartilage are chondrocytes and their ECM. They are crucial for maintaining cartilage homeostasis and are frequently compromised in the malfunctioning cartilage associated with OA.^13,14^ Previous studies have demonstrated that the upregulated advanced glycation end-products (AGEs) in joint aging and increased lysyl oxidase (LOX) in cartilage injuries both accelerated matrix stiffening in vitro, due to the matrix-crosslinking and loss of glycosaminoglycans (GAGs).^15,16^ In fact, it has been demonstrated that matrix stiffness can directly regulate the behavior and phenotype of cells in vitro.^17,18^ Since chondrocytes represent the only cell type in articular cartilage, they can cause cartilage damage when they exhibit metabolic dysfunction.^19^ OA cartilage lesions display higher senescent chondrocyte levels than young, healthy cartilage, suggesting a strong correlation between chondrocyte senescence and OA severity.^20^ Interestingly, the removal of senescent cells from mouse joints not only prevents disease progression but also maintains tissue architecture.^21^ However, the role of ECM stiffness in the regulation of chondrocyte senescence and osteoarthritis progression is unclear. In addition, specific molecular targets directly related to ECM stiffness in osteoarthritis chondrocytes were not revealed. Therefore, the studies are needed to determine the chondrocyte phenotype under stimulation of ECM stiffening and the molecular targets for maintaining cartilage homeostasis.

In this study, we sought to clarify the biomechanics of ECM and the role of chondrocyte mechanotransduction in the initiation and progression of OA. Given the immediacy of ECM-cell contact, it is hypothesized that ECM stiffening is one of the earliest events of OA initiation, leading to mechanical chondrocyte regulation. Therefore, the ECM stiffness changes in a clear time range after injury, and its chondrocyte phenotype regulation is investigated. We found that ECM stiffening stimulated senescence in chondrocyte and in articular cartilage, and identified that histone deacetylase 3 (*HDAC3*) is a key factor in the regulation of chondrocyte senescence phenotype by matrix stiffness. Matrix stiffening downregulates *HDAC3* to activate phosphatase and tensin homolog-induced kinase 1 (*PINK1*) and *Parkin* (*PINK1*/*Parkin)* mediated mitophagy, thereby stimulating chondrocyte senescence and accelerating the initiation and progression of osteoarthritis. Targeting *HDAC3* or *PINK1*/*Parkin* signaling may represent a novel therapeutic approach for OA treatment.

## Result

### ECM stiffening induces chondrocyte senescence in OA patients and model mice

**Fig. 1:**
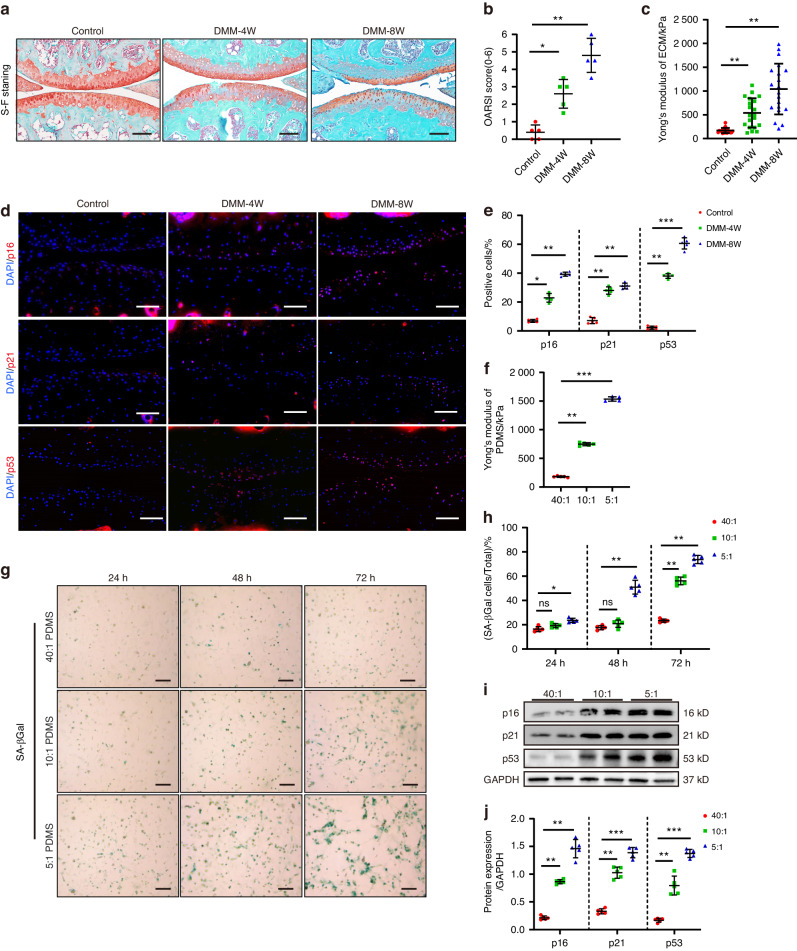
ECM stiffening induced chondrocyte senescence in vitro and in mice. **a** Representative images of safranin O/fast green staining of controls and mice at 4 and 8 weeks after DMM surgery. Scale bars: 100 µm. **b** Quantitative analysis of the OARSI scale (*n* = 5). **c** Measurement of matrix stiffness (Young’s modulus) for controls and mice at 4 and 8 weeks after DMM surgery using atomic force microscopy (AFM) (*n* = 20). **d** Representative images of immunofluorescence of *p16*^*INK4a*^, *p21*, *p53* in chondrocytes of controls and mice at 4 and 8 weeks after DMM surgery. Scale bars: 50 µm. **e** Quantification of *p16* ^*INK4a*^, *p21*, *p53*-positive chondrocytes as a proportion of the total chondrocytes of control and DMM mice (*n* = 5). Data are shown as mean ± SD. **f** Measurement of matrix stiffness (Young’s modulus) for 40:1, 10:1 and 5:1 PDMS using atomic force microscopy (AFM) (*n* = 5). **g**, **h** Representative images and quantification of SA-βGal staining in primary chondrocytes cultivated on PDMS substrates of different stiffness (40:1,10:1, 5:1) for 24, 48, and 72 h (*n* = 5). Scale bar: 50 µm. **i**, **j** Western Blotting analysis of *p16*^*INK4a*^, *p21* and *p53* expression in mouse primary chondrocytes cultivated on 40:1,10:1, 5:1PDMS for 48 h (*n* = 5). *P < 0.05, **P < 0.01, ***P < 0.001. ns not significant; Con control, DMM destablization of the medial meniscus, DAPI 4’,6-diamidino-2-phenylindole, OARSI Osteoarthritis Research Society International, 40:1, 10:1, 5:1, 40:1, 10:1, 5:1 polydimethylsiloxane (PDMS) substrates

To examine the mechanical properties of cartilage ECM during OA in mice, this study used atomic force microscopy (AFM) to measure the ECM stiffness (Young’s modulus) of mouse medial tibial cartilage in a mechanical load inducted OA model (destabilization of the medial meniscus, DMM), since it reflected the most common area of human KOA.^22^ The results showed that the OA model mice displayed obvious surface fibrosis and structural cartilage defects at 4 weeks (4 W) and 8 weeks (8 W) (Fig. 1a, b), with cartilage stiffness values of 537.9 kPa and 1 040.5 kPa, respectively, which were about three- and sixfold higher than in normal cartilage (Fig. 1c). Consistent with these results, the ECM stiffness also increased in human OA cartilage samples (Fig. S1A). Therefore, increased ECM stiffness was positively correlated with the degree of cartilage damage. In addition, the number of stained articular chondrocytes for *p16*^*INK4a*^, *p21*, and *p53* increased markedly in the DMM groups (Fig. 1d, e), which was characteristic of senescent cells.^23,24^ This suggests an association between ECM stiffness and the cartilage senescent phenotype.

To further evaluate the direct effect of different degrees of ECM stiffening on the chondrocyte phenotype, primary mouse chondrocytes were implanted on polydimethylsiloxane (PDMS) substrates at 40:1(≈180 kPa), 10:1(≈750 kPa), 5:1(≈1 515 kPa) since they overlapped with the physiological and pathological ECM stiffness range of mouse and human knee cartilage in this study (Fig. 1f). Compared with the chondrocytes cultivated on soft substrates, those grown on stiffness substrates exhibited senescence, phenotypes and slow cell proliferation, while propagation almost ceased entirely 48 h after inoculation. The chondrocytes cultivated on 5:1 PDMS substrates exhibited decreased activity 72 h after inoculation (Fig. S1B). The number of senescence-associated β-galactosidase (SA-βGal) -stained cells, a classic indicator of senescence, increased in a time-dependent manner after culturing on stiffness substrates (Fig. 1g, h). In addition, stiffness substrates increased the *p16*^*INK4a*^, *p21* and *p53* protein expression in the primary mouse chondrocytes (Fig. 1i, j), which was consistent with the findings regarding the proteins extracted from the articular cartilage of OA patients (Fig. S1C, D). Furthermore, the chondrocyte senescence phenotype regulation by matrix stiffness was consistent with the degree of cartilage component degradation, *Col2a1* expression and upregulated *MMP13* expression in the primary chondrocytes inoculated onto a stiffness matrix (Fig. S1E, F). These results demonstrated that ECM stiffening accelerated chondrocyte senescence in vitro and in articular cartilage, suggesting a potential mechanism in OA pathogenesis and development.

### Reduced chondrocyte *HDAC3* is associated with ECM stiffening in the articular cartilage of OA patients and aged mice

**Fig. 2:**
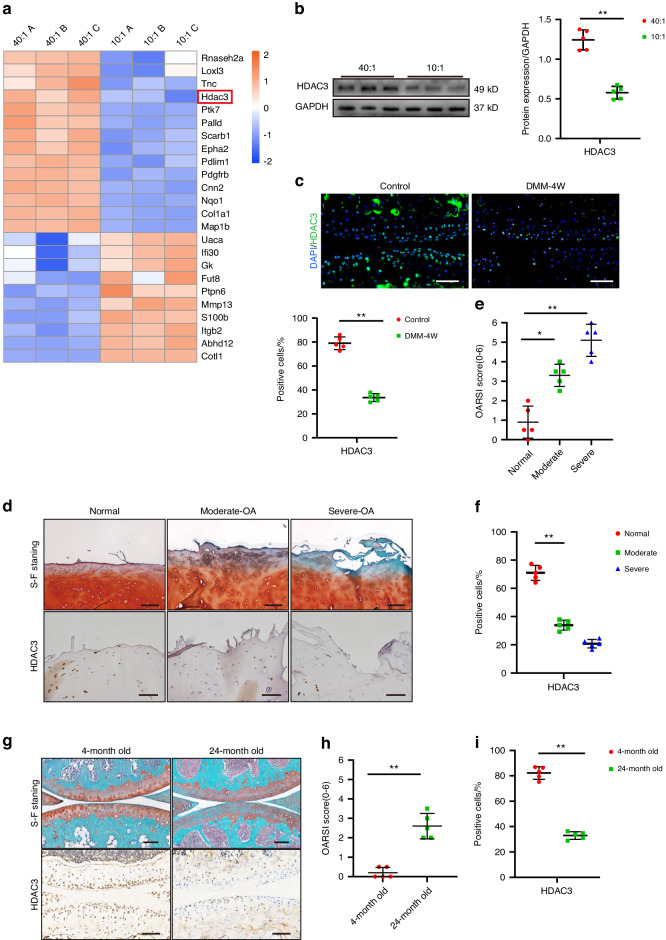
Chondrocyte *HDAC3* is reduced by ECM stiffening and is decreased in the articular cartilage of OA patients. **a** Heatmap of mechanosensitive proteins differentially present in mouse chondrocytes under physiological and pathological stiffness stimuli identified by quantitative proteomics. **b** Western Blotting analysis of *HDAC3* expression in mouse primary chondrocytes cultivated on Physiologic and Pathologic stiffness of PDMS substrates (*n* = 5). **c** Representative images and quantification of immunofluorescence of *HDAC3* in chondrocytes of controls and mice at 4 weeks after DMM surgery. Scale bars: 50 µm. **d** Representative images of safranin O/fast green and IHC staining of *HDAC3* in articular cartilage from normal and OA patients in moderately damaged and severely damaged. Scale bars: 50 µm. **e** Quantification of the OARSI scale based on staining results in (**d**) (*n* = 5). **f** Quantification of *HDAC3*-positive chondrocytes based on staining results in (**d**) (*n* = 5). **g** Representative images of safranin O/fast green and IHC staining of *HDAC3* in chondrocytes of mice aged 4 and 24 months. Scale bars: 100 µm. **h** Quantification of the OARSI scale based on staining results in (**g**) (*n* = 5). **i** Quantification of *HDAC3*-positive chondrocytes based on staining results in (**g**) (*n* = 5). *P < 0.05, **P < 0.01. ns not signicicant. *OA* osteoarthritis; *DAPI* 4’,6-diamidino-2- phenylindole; OARSI Osteoarthritis Research Society International. 40:1, 10:1, 40:1, and 10:1 polydimethylsiloxane (PDMS) substrates

The ECM stiffening mechanism behind chondrocyte senescence stimulation was subsequently investigated. Quantitative proteomic analysis was performed to map the protein profiles of the primary mouse chondrocytes exposed to physiological and pathological stiffness substrate stimulation. Of the 24 differentially expressed proteins regulated by mechanical stimuli, the significant *HDAC3* downregulation stood out (Fig. 2a). *HDAC3* belongs to the histone deacetylase family and is essential for proper endochondral ossification during development and aging-related bone marrow obesity.^25^ However, its role in the chondrocyte senescence stimulated by ECM stiffening remains unclear. Western blotting confirmed significantly decreased *HDAC3* levels in the chondrocytes stimulated by ECM stiffening (Fig. 2b). Consistent with this result, lower *HDAC3* mRNA and protein levels were evident in primary human chondrocytes cultivated on stiffness substrates (Fig. S2A–C). In addition, immunofluorescence (IF) staining showed substantially decreased *HDAC3* levels in the cartilage of OA mice suffering from ECM stiffening (Fig. 2c).

To determine whether the chondrocyte response to ECM stiffening stimuli involved decreased or increased *HDAC3* protein production, primary mouse chondrocytes were treated with cycloheximide (CHX) (50 µm) to block new protein synthesis or MG132 (10 µm) to inhibit proteolysis during culturing on ECM stiffening substrates for 12 h. The HDAC3 expression level decreased significantly after treatment with CHX alone, while no significant difference was evident in the *HDAC3* protein level after ECM stiffening stimulation, regardless of whether CHX treatment was employed (Fig. S2D, E). In addition, although proteolysis inhibition by MG132 increased the *HDAC3* protein level in the control cells, it could not prevent the decrease in the *HDAC3* protein level stimulated by ECM stiffening (Fig. S2F, G). These results indicated that the *HDAC3* loss caused by ECM sclerosis was mainly due to reduced *HDAC3* protein synthesis.

In addition, the reduced *HDAC3* expression in OA patients was associated with cartilage damage, which was confirmed by immunohistochemical (IHC) staining and western blotting (Fig. 2d–f and Fig. S2H, I). Furthermore, lower *HDAC3* expression was observed in aged mice. Compared with 6-month-old mice, the number of *HDAC3*-positive cells decreased significantly in the articular cartilage of 24-month-old mice (Fig. 2g–i). Taken together, these results suggest that *HDAC3* may play a role in the association between ECM stiffening and chondrocyte senescence during OA development.

### ECM stiffening activates *PINK1*/*Parkin-mediated* mitophagy, chondrocyte senescence, and joint degeneration in mice

**Fig. 3:**
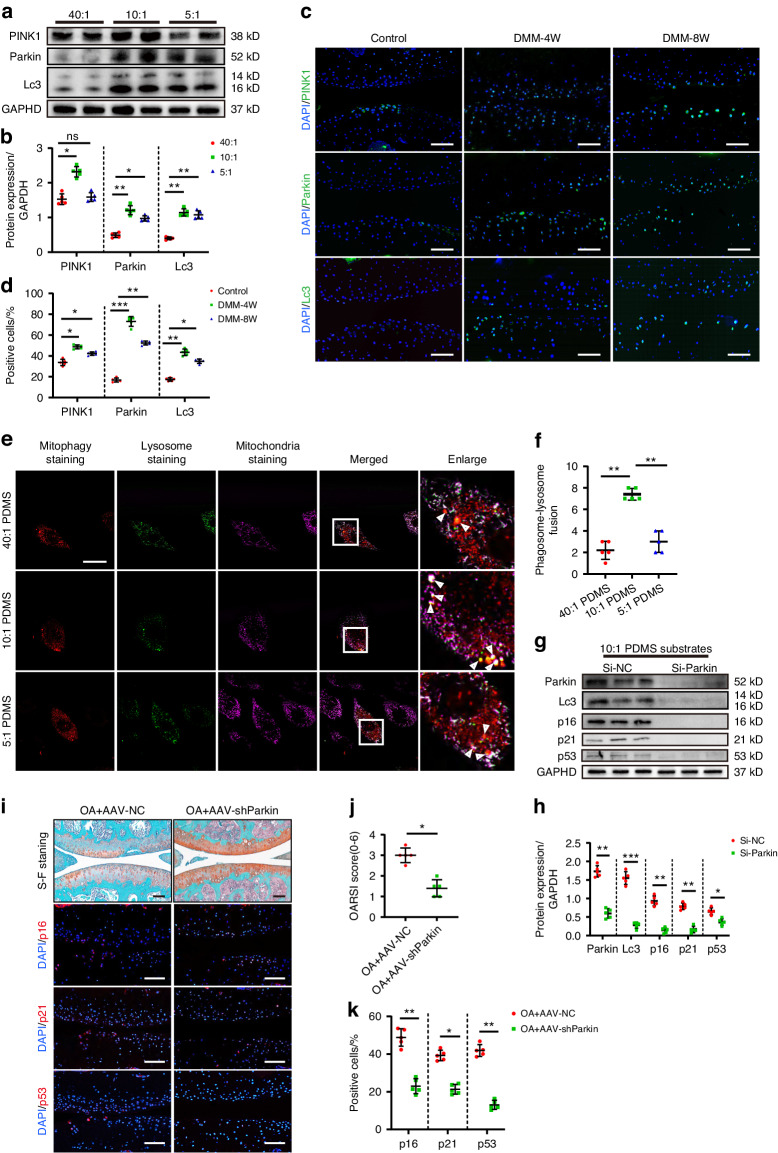
ECM stiffening activates *PINK1*/*Parkin* mediated mitophagy which its inhibition delays chondrocyte senescence and joint degeneration in mice. **a**, **b** Western Blotting analysis of *PINK1*, *Parkin,* and *Lc3* expression in mouse primary chondrocytes cultivated on 40:1, 10:1, and 5:1 PDMS substrates (*n* = 5). **c** Representative images of immunofluorescence of *PINK1*, *Parkin*, *Lc3* in chondrocytes of controls and mice at the end of 4 weeks and 8 weeks after DMM surgery. Scale bars: 50 µm. **d** Quantification of *p16*^*INK4a*^, *p21*, *p53*-positive chondrocytes based on staining results in (**c**) (*n* = 5). **e** Representative images of mitochondria staining, lysosome staining and mitophagy staining in mouse primary chondrocytes cultivated on 40:1, 10:1, and 5:1 PDMS substrates. Scale bars: 5 µm. **f** Quantification of phagosome-lysosome fusion in chondrocytes based on staining results in (**e**) (*n* = 5). **g**, **h** Western Blotting analysis of *Parkin*, *Lc3*, *p16*^*INK4a*^, *p21,* and *p53* in mouse primary chondrocytes which transfected si-NC or si-*Parkin* cultivated on 10:1 PDMS substrates (*n* = 5). **i** Representative images of safranin O/fast green and immunofluorescence staining of *p16*^*INK4a*^, *p21,* and *p53* in the cartilage of mice intra-articularly injected with AAV-NC or AAV-sh*Parkin* after DMM surgery. Scale bars: 50 µm. **j** Quantification of the OARSI scale based on staining results in (**i**) (*n* = 5). **k** Quantification of *p16* ^*INK4a*^, *p21*-positive chondrocytes based on staining results in (**i**) (*n* = 5). *P < 0.05, **P < 0.01, ***P < 0.001. ns not significant; Con control, DMM destablization of the medial meniscus, DAPI 4’,6-diamidino-2-phenylindole, OARSI Osteoarthritis Research Society International, 40:1, 10:1, 5:1, 40:1, 10:1, 5:1 polydimethylsiloxane (PDMS) substrates

Further proteomic analysis showed that the mitochondrial compositional differences were highly correlated with those of ECM, actin fiber bundles, and stress fibers (Fig. S3A). Although studies have shown that excessive mitophagy contributes to many pathological conditions,^26–28^ the regulatory role and mechanism of ECM stiffening in mitophagy have not yet been clarified. Western blotting showed a significantly higher mitophagy level in the chondrocytes cultivated on pathological stiffness substrates than those on physiological stiffness substrates (Fig. 3a, b). However, the level of mitophagy was not completely consistent with the substrate stiffness. Compared with the moderate degeneration group, the chondrocyte mitophagy level was slightly reduced in the severe degeneration group, which was consistent with the observation of the chondrocytes in DMM modeled OA mice and OA patients (Fig. 3c, d and Fig. S3B, C). The mitochondrial proteins were directly extracted from the chondrocytes for validation, yielding the same results (Fig. S3D, E). The lysosomes and mitochondria were localized in the chondrocytes to further determine the mitophagy variation at different pathological stiffness stimuli. The damaged mitochondria fused with the lysosomes during the induction of mitophagy, releasing bright fluorescence. Consistent with previous results, the chondrocytes in the moderate degeneration group contained the most mitophagy vesicles (Fig. 3e, f). In addition, the moderate degeneration group exhibited the most significant decline in mitochondrial membrane potential, marking the early onset of mitochondrial damage (Fig. S3F, G). It is speculated that the excessive number of damaged mitochondria during high-stiffness stimulation leads to an absolute shortage of intracellular mitochondria, decreasing mitophagy activation. Electron microscopy confirmed the hypothesis. The number of damaged mitochondria in the severe degeneration group was significantly higher than in the moderate degeneration group, while the number of lysosome-encapsulated mitochondria decreased (Fig. S3H, I). In addition, the effect of ECM stiffening on mitochondrial homeostasis was tested. The results showed that ECM stiffness reduced the chondrocyte ability to generate mitochondria, as evidenced by a decrease in the *MFN1* and *MFN2* mitochondrial fusion markers in response to higher substrate stiffness, as well as the upregulation of the *DRP1* and *FIS1* mitochondrial division markers (Fig. S4A, B). Consistent with expectations, the level of mitochondrial fission in chondrocytes from the severe degeneration group was slightly reduced relative to the moderate degeneration group, which remains likely to be attributable to increased mitochondrial dysfunction in response to high stiffness stimulation and an absolute deficit in the number of mitochondria.

Subsequently, the relationship between ECM stiffening-induced mitophagy and chondrocyte aging, as well as OA progression, was investigated. Considering the substantial decline in the number of mitochondria cultivated on high-stiffness substrates, it may not be possible to salvage mitochondrial loss via in vitro and in vivo regulation. Next, this work focuses on alleviating mitochondrial homeostasis disorders in the moderate degeneration group. *Parkin* recruitment and activation are necessary for *Parkin*-dependent mitophagy. After silencing *Parkin* expression with small-interfering RNA (siRNA), the *p16*^*INK4a*^, *p21*, and *p53* protein expression of the chondrocytes cultivated on pathological stiffness substrates decreased significantly (Fig. 3g, h). Treatment with cyclosporin A (10 μmol/L, Macklin), an inhibitor of mitochondrial autophagy, similarly attenuates the senescent phenotype of chondrocytes (Fig. S4C, D). Moreover, *Parkin* interference reduced the positive SA-Gal staining rate of the chondrocytes cultivated on pathological stiffness matrix substrates (Fig. S4E, F).

In vivo, Adeno-associated virus interfering *Parkin* (AAV-sh*Parkin*) and comparable amounts of an AAV-negative control were injected intraarticularly once a week from 3 days after DMM surgery. As expected, AAV-sh*Parkin* effectively alleviated OA development in mice by reducing cartilage damage and proteoglycan loss (Fig. 3i, j). Importantly, AAV-sh*Parkin* also reduced the number of *p16*^*INK4a*^, *p21* and *p53* positive chondrocytes in the articular cartilage (Fig. 3i, k). Together, these data suggest that inhibiting *PINK1*/*Parkin*-dependent mitophagy can alleviate the chondrocyte senescence and OA progression caused by ECM stiffening.

### *HDAC3* loss activates *PINK1*/*Parkin* signaling to promote chondrocyte senescence and OA progression

**Fig. 4:**
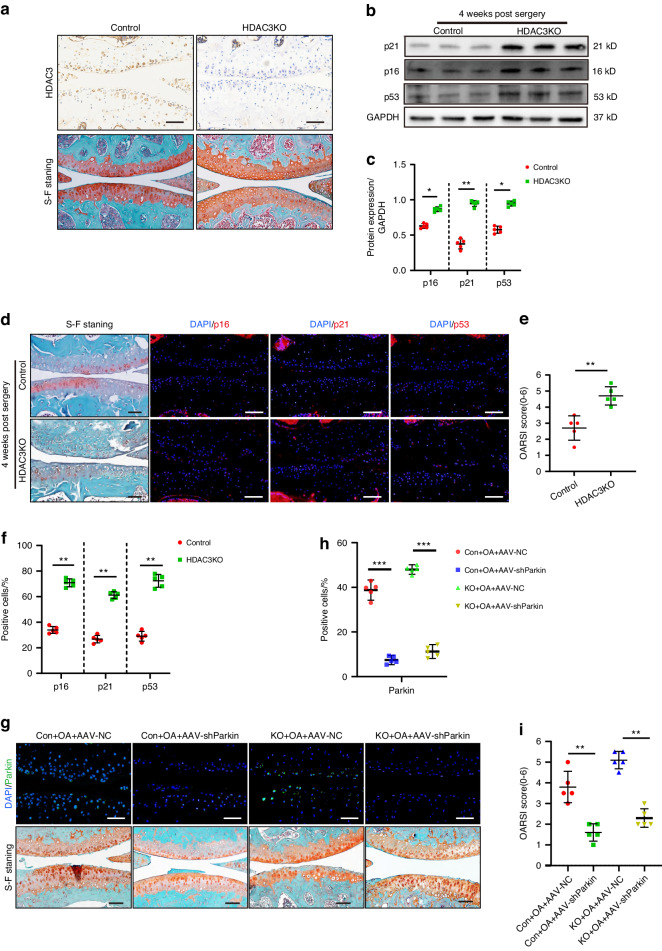
Loss of *HDAC3* activates *PINK1*/*Parkin* signaling to promote chondrocyte senescence and OA progression. **a** Representative images of IHC and safranin O/fast green staining of *HDAC3* in articular cartilage of *HDAC3*KO and Control mice at 3 months old. Scale bar: 50 µm. **b**, **c** Western Blotting analysis of *p16*^*INK4a*^, *p21*, *p53* in the cartilage of *HDAC3*KO and Control mice after DMM surgery (*n* = 5). **d** Representative images of safranin O/fast green and immunofluorescence staining of *p16*^*INK4a*^, *p21*, and *p53* in *HDAC3*KO and Control cartilage of mice after DMM surgery. Scale bars: 50 µm. **e** Quantification of the OARSI scale based on staining results in (**d**) (*n* = 5). **f** Quantification of *p16*^*INK4a*^, *p21*, and *p53*-positive chondrocytes based on staining results in (**d**) (*n* = 5). **g** Representative images of safranin O/fast green and immunofluorescence staining of *Parkin* in *HDAC3*KO and Control cartilage of mice which intra-articularly injected with AAV-NC or AAV-sh*Parkin* after DMM surgery. Scale bar: 50 µm. **h** Quantification of *Parkin*-positive chondrocytes based on staining results in (**g**) (*n* = 5). **i** Quantification of the OARSI scale based on staining results in (**g**) (*n* = 5). *P < 0.05, **P < 0.01. DMM destablization of the medial meniscus, DAPI 4’,6-diamidino-2-phenylindole, OARSI Osteoarthritis Research Society International, KO knockout, AAV-sh*Parkin* adenovirus expressing small hairpin Parkin, AAV-NC negative control

To determine the relationship between chondrocyte *HDAC3* and mitophagy as well as OA progression, knockout mice (*HDAC3*KO) with a conditionally deleted *HDAC3* gene in the chondrocytes were produced by crossing *HDAC3*^flox/flox^ mice with Col2a1-cre mice, The genotypes were determined via PCR (Fig. S5A–C). The body lengths of 8-week-old *HDAC3*KO mice were marginally shorter than those of the control group, and their body weight was noticeably lower, suggesting that their growth of was slower (Fig. S5D–F). In addition, IHC staining further confirmed *HDAC3* ablation in the articular chondrocytes of the *HDAC3KO* mice (Fig. 4a).

**Fig. 5:**
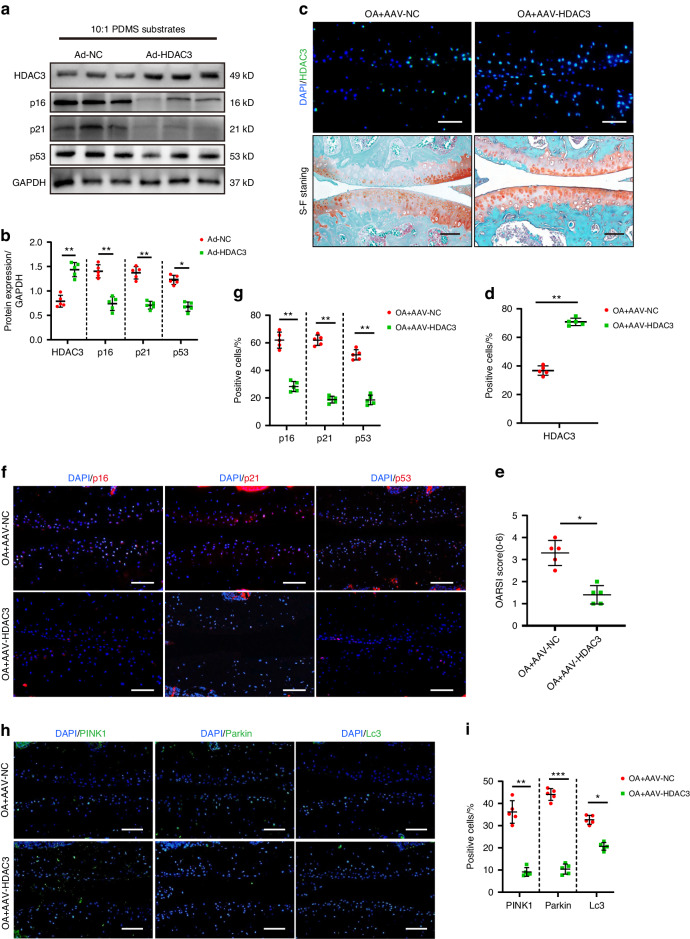
*HDAC3* Overexpression delays chondrocyte aging and OA progression. **a**, **b** Western Blotting analysis of *HDAC3*, *p16*^*INK4a*^, *p21*, and *p53* expression in the primary chondrocytes of mice which transfected adenovirus containing *HDAC3* (Ad-*HDAC3*) or Ad-NC cultivated on 10:1 PDMS substrates (*n* = 5). **c** Representative images of safranin O/fast green and immunofluorescence staining of *HDAC3* in cartilage of mice intra-articularly injected with AAV-NC or AAV-*HDAC3* after DMM surgery. Scale bars: 50 µm. **d** Quantification of *HDAC3*-positive chondrocytes based on staining results in (**c**) (*n* = 5). **e** Quantification of the OARSI scale based on staining results in (**c**) (*n* = 5). **f** Representative images of immunofluorescence staining of *p16*^*INK4a*^, *p21*, and *p53* in cartilage of mice intra-articularly injected with AAV-NC or AAV-*HDAC3* after DMM surgery. Scale bars: 50 µm. **g** Quantification of *p16*^*INK4a*^, *p21*, and *p53*-positive chondrocytes based on staining results in (**f**) (*n* = 5). **h** Representative images of immunofluorescence staining of *PINK1*, *Parkin,* and *Lc3* in cartilage of mice intra-articularly injected with AAV-NC or AAV-*HDAC3* after DMM surgery. Scale bars: 50 µm. **i** Quantification of *PINK1*, *Parkin* and *Lc3*-positive chondrocytes based on staining results in (**h**) (*n* = 5). *P < 0.05, **P < 0.01, ***P < 0.001. OA osteoarthritis, DAPI 4’,6-diamidino-2-phenylindole, OARSI Osteoarthritis Research Society International, AAV-*HDAC3* adenovirus expressing small hairpin *HDAC3*, AAV-NC negative control

When cultured on physiological stiffness substrates, chondrocytes from *HDAC3*KO mice and controls showed no appreciable changes in senescence phenotype (Fig. S6A, B). However, when cultured on pathological stiffness substrates, the primary chondrocytes with *HDAC3* gene deletion enhanced the senescence phenotype. It is shown that the protein upregulation of *p16*^INKA4^, *p21*, *p53* and enhancement of SA-β-galactosidase staining (Fig. 4b, c and Fig. S6C, D). The data suggest that *HDAC3* deficiency alone is not sufficient to induce chondrocyte senescence in the absence of mechanical stimuli. Silencing the Parkin expression in the *HDAC3*KO chondrocytes alleviated the aging phenotype resulting from *HDAC3* deletion (Fig. S6E, F). This suggests that *HDAC3* gene deletion promotes chondrocyte senescence by activating *PINK1*/*Parkin*-dependent mitophagy. *HDAC3* gene deletion also encouraged chondrocyte senescence and OA progression in vivo. Compared with the control group, *HDAC3* deletion accelerated the occurrence of experimental OA, showing significant cartilage erosion, as well as proteoglycan and cell density loss in the articular cartilage, which was further verified using the scale analysis of the International Osteoarthritis Research Institute (OARSI) (Fig. 4d, e). Moreover, the number of positive *p16*^*INK4a*^, *p21*, and *p53* cells in the joint chondrocytes of the *HDAC3*KO mice was significantly higher than that of their control group offspring (Fig. 4d, f). Consistent with previous results, *Parkin* inhibition alleviated articular cartilage erosion in the *HDAC3*KO DMM model mice (Fig. 5g–i). In summary, these results indicate that the absence of *HDAC3* in the chondrocytes accelerates the cell and cartilage aging caused by ECM stiffening.

### *HDAC3* overexpression delays chondrocyte aging and OA progression

The primary mouse chondrocytes cultured on pathological stiffness substrates were treated with and without *HDAC3* containing adenovirus (Ad-*HDAC3*) for 24 h. The *p16*^*INK4a*^, *p21*, and *p53* levels decreased significantly in the Ad-*HDAC3*-treated chondrocytes, while the number of positively SA-β-Galactosidase-stained chondrocytes decreased (Fig. 5a, b and Fig. S6G, H), Indicating that *HDAC3* addition alleviated the chondrocyte aging caused by ECM stiffening. Next, starting 3 days after DMM surgery, AAV-*HDAC3* and an equal amount of an AAV-negative control were injected weekly into the joints of the mice. IF showed that AAV-*HDAC3* intra-articular injection mainly affected the articular cartilage. Additionally, significantly higher *HDAC3* expression was evident in the deep-layer chondrocytes of the AAV-*HDAC3*-treated mice, confirming that AAV successfully transuded *HDAC3* overexpression (Fig. 5c, d). The results showed that AAV-*HDAC3* effectively alleviated chondrocyte aging and OA development in mice by decreasing the number of positive *p16*^*INK4a*^*, p21*, and *p53* cells (Fig. 5f, g). Moreover, the hypertrophic chondrocyte differentiation, cartilage destruction, and proteoglycan loss were reduced in the tibial cartilage of the AAV-*HDAC3*-treated mice (Fig. 5c, e). As expected, *HDAC3* overexpression significantly reduced *PINK1/Parkin*-dependent mitophagy in the mouse chondrocytes (Fig. 5h, i). In summary, these findings suggest that *HDAC3* delays ECM stiffening-induced chondrocyte senescence and OA progression.

### HDAC3 affects mitophagy via epigenetic Parkin regulation

**Fig. 6:**
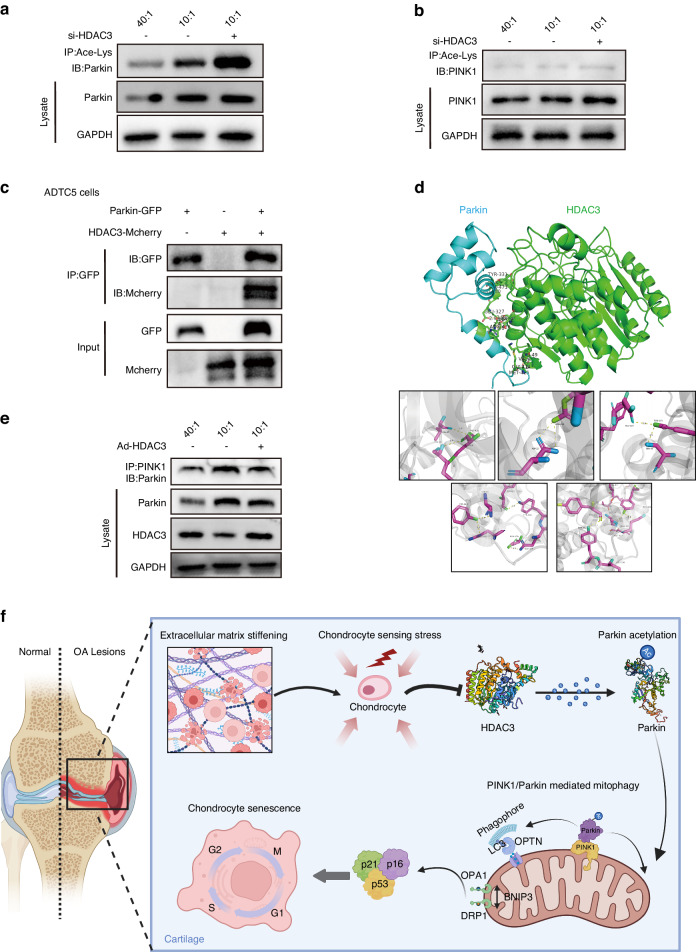
*HDAC3* affects mitophagy through epigenetic regulation of *Parkin*. **a** Acetylation of endogenous *Parkin* in chondrocytes transfected si-NC or si-*HDAC3* cultivated on 40:1 and 10:1 PDMS substrates. *Parkin* acetylation was analyzed by immunoprecipitation with an anti-acetyl-lys antibody followed by western blotting for *Parkin*. **b** Acetylation of endogenous *PINK1* in chondrocytes transfected si-NC or si-*HDAC3* cultivated on 40:1 and 10:1 PDMS substrates. *PINK1* acetylation was analyzed by immunoprecipitation with an anti-acetyl-lys antibody followed by western blotting for *PINK1*. **c** Mcherry and GFP were immunoprecipitated from ATDC5 cells after transfection with both plasmids containing *HDAC3*-Mcherry and *Parkin*-GFP. The presence of Mcherry and GFP in the immunoprecipitates was evaluated by immunoblotting. **d** Top: optimized binding modes with the lowest binding energy generated by ZDock, and key residues for interaction between mouse *HDAC3* and *Parkin*. Down: magnified view of boxed area. **e** Cell lysates of chondrocytes which transfected adenovirus containing HDAC3 (Ad-HDAC3) or Ad-NC cultivated on 40:1 and 10:1 PDMS substrates were subjected to immunoprecipitation with anti-*PINK1* antibody followed by immunoblotting to detect *Parkin*. **f** Schematic diagram representing molecular pathways in which ECM stiffening induces OA development through *HDAC3*. IB immunoblotting, IP immunoprecipitate

Next, this study explored the HDAC3 regulation of chondrocyte mitophagy. HDAC3 is mainly considered an epigenomic regulator of deacetylate histones, which in turn controls the transcription of the various genes required for development and physiology.^29^ Recent studies have shown that HDAC3 can drive non-histone deacetylation outside the nucleus.^30^ It is well known that protein acetylation is mainly responsible for transcriptional gene regulation. First, the transcriptional regulation of the mitophagy-related genes by *HDAC3* was investigated. When stimulated by pathological ECM stiffening, the *Prkn* chondrocyte difference was more obvious in the *HDAC3*KO mice than in the control group (Fig. S6I). Subsequently, the Parkin acetylation level was investigated via immunoprecipitation. The *HDAC3* loss caused by ECM stiffening activated *Parkin* acetylation, which increased further after *HDAC3* knockdown (Fig. 6a), showing that *HDAC3* significantly affected *Parkin* acetylation regulation. However, as a protein equally critical for mitochondrial autophagy initiation, no significant differences were evident in the acetylation level of *PINK1* affected by sclerosis or *HDAC3* knockdown (Fig. 6b). In addition, the interaction between the *HDAC3* and Parkin in ATDC5 cells was determined (Fig. 6c). To help identify which *HDAC3* and *Parkin* sites participated in this binding event, a 3D *HDAC3-Parkin* complex structure was modeled using the ZDOCK protein-docking algorithm (Fig. 6d). Protein docking results confirmed that there were multiple interaction sites on the interaction surface of *HDAC3* and *Parkin*, including seven hydrogen bonds generated by groups such as lys49 and gly414, and the binding energy between *HDAC3* and *Parkin* protein was −17.9 kcal/mol, indicating that they had strong affinity activity.

As an E3 ubiquitin ligase, *Parkin* mediates mitophagy downstream of *PINK1*.^31^ The effect of acetylation modification on the interaction between *Parkin* and its upstream *PINK1* protein was investigated. As expected, culturing on a stiffness substrate increased the level of interacting *Parkin* in the immunoprecipitated *PINK1* protein complex, while *HDAC3* overexpression decreased the interaction between *Parkin* and *PINK1* (Fig. 6e). The above results indicate that *HDAC3* regulated *Parkin* acetylation is involved in the induction of mitophagy.

## Discussion

The mechanical properties of ECM regulate a variety of biological cell phenotypes. *HDAC3* was found to be a critical component in the relationship between ECM mechanical properties and chondrocyte senescence. ECM stiffening during the OA process stimulated chondrocyte senescence in vitro and in mouse articular cartilage. Furthermore, ECM stiffening stimuli downregulated *HDAC3*, which increased *Parkin* acetylation and activated *Parkin*-dependent mitophagy, promoting chondrocyte senescence and cartilage degradation. Therefore, *HDAC3* supplementation and *Parkin*-dependent mitophagy inhibition are potential targets for OA treatment (Fig. 6f).

This study showed that OA cartilage exhibited a stiffer cartilage matrix than normal cartilage, while the cartilage matrix stiffness increased with the OA severity. Changes in the biomechanical properties of the cartilage matrix can be attributed to changes in the cartilage composition and structure. Cartilage, mainly composed of collagen fibers, proteoglycans, and water, supports weight in joints. The collagen fibers are exposed during OA development, while water and proteoglycan loss produce cross-linked collagen fibers that confer tensile strength to tissues stiffer than intact normal cartilage. To biosimulate the effect of matrix stiffness on chondrocytes, a matrix stiffness model was established based on PDMS to examine the interaction between chondrocytes and ECM stiffness. Various studies have examined matrix stiffness in cell culture systems.^32,33^ In cancer, matrix stiffness can promote tumor growth by directly stimulating or inducing exosome secretion.^17^ In addition, previous research showed that matrix stiffness guided stem cell lineage specification and proliferation.^34^ The present study found that pathological matrix stiffness stimulation accelerated chondrocyte and cartilage aging in mice, indicating interaction between the biomechanical and biological environments during OA.

*HDAC3* is widely regarded as an epigenetic cell regulator. It cooperates with the silencing mediator of retinoic acid and thyroid hormone receptor and nuclear receptor corepressor to promote histone deacetylation and targets a variety of pathways, such as gene transcription, cell development, cell cycle, and tumorigenesis. In addition, evidence indicates *HDAC3* mechanosensitivity.^29^ Previous studies showed that abnormal matrix stiffness in liver cirrhosis changed *HDAC3* expression and the organization of its cytoskeleton.^35^ Another study indicated that HDAC3 was essential during shear-induced stem cell differentiation.^36^ However, no studies are available on the role of HDAC3 in chondrocyte senescence regulation and OA progression. In this study, mass spectrometric proteomic analysis showed that HDAC3 differed significantly in proteins regulated by mechanical stimulation. Subsequent experiments also verified that HDAC3 was significantly downregulated by matrix sclerosis stimulation in chondrocytes in vivo and in vitro, while its deletion caused chondrocyte senescence and OA deterioration. In addition, HDAC3 deletion enhanced the chondrocyte senescence phenotype in the articular cartilage of DMM OA mice, while in vivo HDAC3 overexpression alleviated chondrocyte senescence and OA progression. However, no significant changes were evident in the chondrocyte senescence phenotype in the HDAC3KO mice and controls, suggesting that HDAC3 deficiency alone was insufficient for inducing chondrocyte senescence in the absence of mechanical stimuli.

The investigation into the role of downstream HDAC3 signaling in regulating chondrocyte senescence and OA development via ECM stiffening was noteworthy since it revealed a strong correlation between the compositional variations of the ECM, actin fiber bundles, and stress fibers and those of the mitochondria. Mitochondria represent the “power source” of cells, producing cellular energy in the form of ATP and participating in various important cellular processes.^37^ Since mitochondrial dysfunction is associated with cellular damage and a variety of diseases, it is essential to maintain mitochondrial homeostasis.^38,39^ Mitophagy is a unique form of autophagy that regulates the turnover of dysfunctional mitochondria and is a key mechanism for maintaining mitochondrial homeostasis.^40^ Considerable evidence indicates an association between mitophagy and OA progression.^41,42^ However, some controversy exists about the role of mitophagy in OA progression. Studies have shown that *Parkin* overexpression reduces mitochondrial ROS and chondrocyte apoptosis by scavenging dysfunctional mitochondria.^43^ In addition, metformin can also activate the *sirt3-PINK1-prkn* signaling pathway to counteract the oxidative stress caused by *IL1B*, as well as the anabolic and catabolic imbalance in chondrocytes.^44^ However, other studies have shown that *PINK1*-mediated mitophagy leads to mitochondrial fragmentation and cell death in human chondrocytes and rats after MIA treatment, while *PINK1* knockout mice with MIA-induced OA exhibit less cartilage damage and pain behavior compared with the control group.^45^ The dual effect of mitophagy on cell survival and function in the above results may be attributed to the mitophagy level in pathological conditions, model intervention differences, and of disease progression stages. This study showed that the *HDAC3* loss stimulated by ECM stiffening significantly activated *Parkin*-dependent mitophagy and accelerated cellular senescence. When stimulated by severe pathological ECM stiffening, the level of *Parkin*-dependent mitophagy decreased slightly compared to the moderate pathological group. It is reasonable to assume that mitophagy is essential for basal mitochondrial turnover and maintenance in physiological conditions and may also play a primary role in initiating various pathological stresses. Mitochondrial clearance activation by excessive stiffness stimulation resulted in a complete lack of mitochondria in the chondrocytes, reducing the relative level of mitophagy. In addition, Parkin interference effectively delayed the chondrocyte senescence phenotype and OA development in the *HDAC3*KO mice, suggesting that Parkin-dependent mitophagy was required for *HDAC3* to affect cartilage aging.

The importance of *HDAC3* in chromatin dynamics and gene expression regulation is well recognized.^46,47^ At the mechanistic level, *HDAC3* regulated mitophagy by affecting *Parkin* acetylation modification. Previous studies have reported the important role of Parkin acetylation in *Parkin*-dependent mitophagy and tumor suppression.^31^ Consistent with this, in this study, *HDAC3* inhibition upregulated the lysine acetylation modification of *Parkin* and activated *Parkin*-dependent mitochondria. These results suggested that the *Parkin* acetylation regulation by *HDAC3* played a vital role in ECM stiffening-induced chondrocyte senescence and cartilage degeneration.

Although the data in this study suggest that *HDAC3-Parkin* signaling is important for chondrocyte senescence and cartilage degeneration, the regulation of mitophagy at an appropriate level requires further investigation. For genetic diseases associated with mitophagy defects, the mutation or deletion of mitophagy-related genes easily causes complex multisystem lesions and neurodegeneration.^48,49^ In addition, although previous studies have shown that part of *HDAC3* is initially located in the cytoplasm, how the remainder approaches mitochondria remain unclear. Therefore, the activity trajectory and function of the *HDAC3* that regulates *Parkin* acetylation require further exploration. Although the practicality and safety of applying *HDAC3-Parkin* signaling for OA treatment requires further verification in future research, the results of this study reveal a causal relationship between ECM stiffening and cartilage aging while determining that *HDAC3* deletion and *Parkin*-dependent mitophagy activation are important for ECM stiffening to regulate cartilage aging. Therefore, targeting *HDAC3-Parkin* signaling may be a novel therapeutic approach for OA treatment. Since the effect of matrix stiffness on OA is still under investigation, this study raises many new hypotheses and expectations. Considering the mechanical sensitivity of chondrocytes to ECM stiffness, modulating the physicochemical properties of ECM to restrict cartilage degeneration using osteochondral scaffolds, which have been extensively investigated to date, is also a feasible topic for future research.^50–52^ Furthermore, determining whether *HDAC3*-dependent ECM stiffening participates in the OA process in any other way or whether *HDAC3-Parkin* signaling modulation alleviates cartilage ECM stiffness during OA will be investigated in continued research.

## Materials and methods

### Human samples

Human osteoarthritis cartilage was obtained from patients undergoing total knee arthroplasty (*n* = 8). Normal control cartilage was obtained from traffic accident patients with no history of arthritis (*n* = 5). Cartilages were excised from the tibial plateau and femoral condyles during total knee replacement surgery. Cartilage samples were fixed in 4% paraformaldehyde, decalcified in 15% EDTA and paraffin-embedded for further histological analysis.

All human samples were obtained from the Third Affiliated Hospital of Southern Medical University, Guangzhou, China. All patients provided informed consent to use their clinical information for scientific research. This study was approved by the Ethics Committee of the Third Affiliated Hospital of Southern Medical University (2024-ER-001).

### Animals

The *col2a1*-cre mice were purchased from Cyagen Biosciences, Jiangsu, China. The primers used are shown below. Forward: CTCTTCGCTATTATTCACCCTCAGCTT; Reverse: CTTGCGAACCTCATCACTCGTTG. Unique product lengths of 431 bp were generated. *HDAC3*^flox/flox^ mice were purchased from GemPharmatech, Jiangsu, China. Both mice were of C57BL/6J background. To generate chondrocyte specific *HDAC3* null mice, col2a1cre mice were bred with *HDAC3*^flox/flox^, and the *HDAC3* chondrocyte-specific null mice were named *HDAC3*KO mice. The offspring mice carrying *HDAC3*^flox/flox^ without CRE were used as the control group. Routine genotyping of tail DNA was performed according to the instructions of GemPharmatech and Cyagen Biosciences. The primers used are shown below. Forward: GTTAATCCGTGGGAGGATATTTTCT; Reverse: CCACTCAAACAAGCATACAGAGAAACA. Unique product lengths of 373 bp were generated.

For mice OA model, all C57BL/6J mice were purchased from the Laboratory Animal Center of Southern Medical University (Guangzhou, China). Twelve-week-old C57BL/6J mice and their littermate control mice underwent medial meniscus destabilization (DMM) surgery to induce osteoarthritis. To reduce the number of animals used, only male mice were used for experiments in this study. 20 mice were randomly assigned to experimental groups. The operative area was shaved, and the mice were selected to be fixed in the supine position with the left hind limb flexed at 90°, and the operative field was fully disinfected. The skin was cut to expose the patellar ligament, and the joint capsule was dissected along the inner edge of the patellar ligament with sharp knife scissors to bluntly clean out the intra-articular adipose tissue, and the medial meniscus was attached to the tibial plateau through the medial meniscus tibial collateral ligament (MMTL). After dissecting the MMTL and flushing the wound, the joint cavity was sutured, the skin wound was closed, and topical amoxicillin was applied to prevent wound infection. In the sham-operated group, only the joint capsule was dissected and then sutured layer by layer. The mice were also observed for anesthesia awakening and wound healing.

Mice were executed at 4 and 8 weeks postoperatively, and knee joint specimens were collected. The knee joints were fixed in 40 g/L paraformaldehyde for 24–48 h, decalcified in 100 g/L EDTA for 1 week, embedded in paraffin wax, sectioned in coronal position at 4 μm intervals, and stained with saffron O/solid green. The samples were scored using the Osteoarthritis Research Society International (OARSI) grading/staging system.

All animals were provided with standard feed and kept in pathogen-free cages with constant temperature and humidity. The circadian rhythm was maintained at 12 h. All animal experiments were approved by the animal protection and use Committee of the Ruiye model animal (Guangzhou, China) Biotechnology Co. (Guangzhou, China) and carried out according to the guidelines and regulations of the Committee (no. RYEth-20231008321).

### Intra-articular delivery of *HDAC3 and shParkin* adeno-associated virus in experimental OA

Adeno-associated virus containing *HDAC3* (AAV-*HDAC3*) (GENECHEM, Shanghai, China) and *shParkin* (AAV-*shParkin*) (GENECHEM, Shanghai, China) was administered to C57BL/6J mice with DMM-induced OA by intra-articular injection performed at 3-, 14-, and 21-day post DMM surgery. Specifically, a longitudinal skin incision was made to visualize the patellar ligament and the patella; then we injected 1 × 10^10^ AAV particles in a 10 µL volume into the knee joint cavity by inserting a small needle into the area underneath the patella of the leg. The control groups were all treated with negative control (AAV-NC) for the same periods. Mice were euthanized at 28 days post-surgery for histological analyses. The knee joints were fixed in 40 g/L paraformaldehyde for 24–48 h, decalcified in 100 g/L EDTA for 1 week, embedded in paraffin wax, and sectioned in coronal position at 4 μm intervals for further histological analysis.

### Histological analyses

Tissue samples were fixed in 4% paraformaldehyde buffered with phosphate-buffered saline (pH 7.4) for 24 h at 4 °C. Specimens were decalcified with 10% EDTA (pH 7.4) for 2 weeks at 4 °C, embedded in paraffin and 4-µm thick sagittal sections were cut. Safranin O/Fast Green staining was performed according to standard protocols. Safranin O/Fast Green staining slides were used to evaluate cartilage degeneration by OARSI scoring system. Each section was assessed by two blinded, independent graders and the mean score was used for statistical analysis.

For OARSI scoring system, we recommend these 0–6 subjective scoring system to apply to all four quadrants of the joint: medial femoral condyle, medial tibial plateau, lateral femoral condyle, lateral tibial plateau. A score of 0 represents normal cartilage, 0.5 = loss of proteoglycan with an intact surface, 1 = superficial fibrillation without loss of cartilage, 2 = vertical clefts and loss of surface lamina, 3 = vertical clefts/erosion to the calcified layer lesion for 1%–25% of the quadrant width, 4 = lesion reaches the calcified cartilage for 25%–50% of the quadrant width, 5 = lesion reaches the calcified cartilage for 50%–75% of the quadrant width, 6 = lesion reaches the calcified cartilage for >75% of the quadrant width. The OA severity is expressed as a maximal score.

### Immunohistochemistry and IF

Specimens were prepared as described previously. Following deparaffinization and rehydration, the sections were soaked in citrate buffer (10 mmol/L citric acid, pH 6.0) for 16–18 h at 60 °C to unmask the antigen for IHC and IF analyses. For IHC analysis, we added 3% hydrogen peroxide for 15 min. The sections were blocked with 1% sheep serum for 1 h at 37 °C and incubated with the primary antibodies (in 1% bovine serum albumin and 0.1% Triton X-100) overnight at 4 °C. For IHC staining, sections were stained with horseradish peroxidase-conjugated secondary antibodies (Proteintech, Hubei, China) and Horseradish Peroxidase Color Development Kit was used to observe the chromogen, with hematoxylin for counterstaining. For IF, sections were stained with Alexa 488 or Alexa 594 dye-labeled secondary antibodies (Thermo Fisher Scientific, MA, USA). Nuclei were labeled with 4, 6-diamidino-2-phenylindole (DAPI; Thermo Fisher Scientific, MA, USA) and images were obtained using a FluoView FV1000 confocal microscope (Olympus, Tokyo, Japan). Sections were randomly coded and scored by two blinded observers for three sections per joint.

### Cell cultures

Primary articular chondrocytes were isolated from 3-day-old C57BL/6J mice knee joint cartilage according to a standard protocol using collagenase II. Primary chondrocytes were cultured in DMEM-F12 with 15% FBS. The medium was changed every day. For most experiments, primary cells were transferred to serum-free DMEM for 24 h before being exposed to stimulation.

### Polydimethylsiloxane (PDMS) substrate preparation

PDMS substrates with different stiffness were prepared from the commercially available Sylgard 184 silicone elastomer kit (Dow Corning, MI, USA) by mixing base and curing agent in varying ratios (base polymer-to-cross-linker ratios, w/w). The pre-polymer mixtures were mixed thoroughly for 5 min, degassed, and poured into culture dishes for spreading. PDMS substrates were cured at 60 °C for 2 h, and the thickness of PDMS substrate was 1 mm. They were then immersed in ethanol for 3 h for sterilization, and washed with 1xPBS three times. We coated PDMS substrates with fibronectin (Millipore, MA, USA) for cell culture according to the manufacturer’s protocol.

### Mechanical characterization

For each joint, two to three sections were chosen from the central, load-bearing region of the tibial cartilage that is in direct contact with condylar cartilage during joint loading (sections 10–30 cut from the medial to lateral ends, following trimming of the first 300 μm thickness at the medial end). The removed tissue was used for frozen sections, on each section, at a distance of about 20–40 μm from the tibial surface. The middle/deep region of the uncalcified cartilage layer of identified one or two regions of interest (ROIs). Within each ROI, AFM (Bruker Co., MA, USA) nanomechanical mapping was performed in a 40 × 40 grid (1 600 indents) using polystyrene microspherical tips up to ≈100 nN maximum indentation force at 10 μm/s effective indentation depth rate. For each indentation, the effective indentation modulus, Eind, was calculated by fitting the entire loading portion of the indentation force-depth (F–D) curve to the finite thickness-corrected Hertz model. All data analyses were performed using NanoScope Analysis2.0 (Bruker Co., MA, USA).

### Senescence associated β-galactosidase (SA-β-Gal) assay

Cytochemical staining for SA-β-Gal was performed using an SA-β-Gal staining kit (Beyotime, Shanghai, China). Following the cell treatment, cytochemical staining for SA-β-Gal was performed at pH 6 according to the manufacturer’s protocol, and the positive cells in four randomly selected fields per treatment were counted (*n* = 5).

### CCK-8 assay for cell viability

The assessment of cell viability was performed using the Cell Counting Kit-8 (Beyotime, Shanghai, China) reagent to evaluate cellular activity. chondrocytes were cultivated on 40:1, 10:1, and 5:1 PDMS substrates. At specified time points, the CCK-8 reagent was added to the culture medium. The cell-CCK-8 mixture was incubated at 37 °C in a cell culture for 1 h. Using a microplate reader (Thermo Fisher Scientific, MA, USA), measure the absorbance of each well at a wavelength of 450 nm.

### Transmission electron microscope

Obtain chondrocytes cultivated on 40:1, 10:1, and 5:1 PDMS substrates. After washing with PBS, chondrocytes were scraped and harvested with 4% glutaraldehyde and centrifuged at 4 °C. The precipitated chondrocytes were continued to fix with 4% glutaraldehyde for another 2 h at room temperature and stored at 4 °C. Electron photomicrographs of the chondrocytes were taken by Scientific Compass Technology Co., Zhejiang, China.

### Western blotting analysis

Tissues and cells were lysed using lysis buffer (62.5 mmol/L Tris-HCl [pH 6.8], 10% glycerol, 2% SDS, 50 mmol/L dithiothreitol, 0.01% bromophenol blue) at 96 °C for 10 min. The samples were separated by SDS-PAGE for 70 min and the proteins were subsequently transferred to membranes (Bio-Rad Laboratories, CA, USA) by the wet transfer method. Each membrane was incubated with primary antibodies overnight at 4 °C on a shaker. Following incubation with specific secondary antibodies, we detected the proteins with an enhanced chemiluminescence kit (Proteintech, Hubei, China). The following primary antibodies were used: *p16*^*INK4a*^ (1:1 000, #18769, Cell Signaling), *p21* (1:1 000, ab188224, Abcam), *p53* (1:1 000 10442-1-AP, Proteintech), *HDAC3* (1:1 000, 10255-1-AP, Proteintech), *GAPDH* (1:5 000, 10494-1-AP, Proteintech), *PINK1* (1:1 000 for Western blot and 1:50 for Immunoprecipitation, #6946, Cell Signaling), *Parkin* (1:1 000, 14060-1-AP, Proteintech), *Lc3* (1:1 000, ab48394, Abcam), *MMP13* (1:1 000, 18165-1-AP, Proteintech), *COL2A1* (1:1 000, #40772, SAB), *Acetylated-lysine* (1:100 for Immunoprecipitation, #9441, Cell Signaling) and *GFP* (1:200 for Immunoprecipitation, 66002-1-Ig, Proteintech).

### Real-time qPCR

Total RNA was isolated from tissue and cell pellets with TRIzol Reagent (Takara Biotechnology, Tokyo, Japan) and reverse transcribed with reverse transcription reagent (Takara Biotechnology, Tokyo, Japan) according to the manufacturer’s protocol. Complementary DNA was used for real-time PCR with SYBR Premix Taq (Vazyme Biotech, Jiangsu, China) in a Light Cycler (Roche Molecular Biochemicals, Basel, Switzerland). Relative quantification of gene expression was performed with the comparative threshold method. Changes in mRNA expression levels were calculated after normalization to values for the GAPDH calibrator gene. The primers used are shown below. *Pink1*(Forward: CGGTCGCACACTGTTCCTCGT; Reverse: CCCTCCAGCAACTGCAAGGTCA), *Prkn* (Forward: TTCCGAATCACCTGACGGTT; Reverse: ATGACTTCTCCTCCGTGGT), *Map1lc3a* (Forward: CCCCAGTAAGATCCCGGTGA; Reverse: ATGATCTTGACCAACTCGCTCA), *Ulk1* (Forward: AAACATCCTGCTGTCCAACCC; Reverse: GCCGCCATCATGTTGCTCT), *Hspd1* (Forward: GTGTGAATTCCAAGATGCCTA, Reverse: TTATGACCAATGGCTTCCGAT), *Sqstm1* (Forward: F: ACCCATCTACAGAGGCTGATCCC; Reverse: CCAGCCGCCTTCATCCGAGA).

### siRNA transfection

Small interfering RNA (siRNA) oligonucleotides against mouse, *Parkin* and nontargeting scrambled control siRNA were purchased from GenePharma, Shanghai, China. Lipofectamine 3000 (Thermo Fisher Scientific, MA, USA) was used to transfect 100 nm siRNA-*HDAC3* and siRNA-*Parkin* in opti-MEM (Thermo Fisher Scientific, MA, USA) following the manufacturer’s protocol. After 24 h of transfection, the cell culture media were replaced with fresh DMEM and incubated for a further for 48 h. The oligonucleotide sequences of *Parkin* siRNA were, forward: 5′GGA AGG AGC UUC CGA AUC ATT 3′; Reverse: 5′- UGA UUC GGA AGC UCC UUC CTT -3′; chondrocytes were transduced with siRNA when the cells were 30%–50% confluent.

### Plasmid constructs and transfection

Mcherry-*HDAC3* was prepared by cloning the corresponding *HDAC3* DNA fragment into the mcherry-pcs2 vector using NheI and xhol restriction sites. EGFP-PRKN was purchased from MIAOLING BIOLOHY, Hubei, China. According to the manufacturer’s instructions, indicated plasmids were transfected using Lipofectamine 3000 (Thermo Fisher Scientific, MA, USA).

### Immunoprecipitation

For immunoprecipitation assays, transduced and treated cells were lysed with RIPA lysis buffer supplemented with a completely EDTA-free protease inhibitor mixture (Beyotime, Shanghai, China) for 30 min on ice. Lysates were removed by centrifugation at 13 000 r/min for 15 min and the supernatant was collected. The protein-containing supernatant was incubated with primary antibody or 1 μg of normal mouse IgG (Proteintech, Hubei, China) as a control for 1 h at 4 °C with rotation. The protein lysates were subsequently incubated with 30 μL of pre-washed protein A-agarose beads (Beyotime, Shanghai, China) for 3 h at 4 °C with gentle rotation. The immunoprecipitates were analyzed by immunoblotting.

### Mitochondrial membrane potential assay (JC-1)

We use JC-1(Beyotime, Shanghai, China) probe to detect the mitochondrial membrane potential. Chondrocytes inoculated in physiological and pathological hardness were resuspended in 0.5 mL of cell culture medium, 0.5 mL of JC-1 staining working solution was added and mixed by inverting several times. Incubate for 20 min at 37 °C in a cell culture incubator. After incubation centrifuge the cells at 600 *g* 4 °C for 3–4 minutes to precipitate the cells. Next, the cells were washed twice with JC-1 staining buffer: 1 mL of JC-1 staining buffer was added to resuspend the cells, centrifuged at 600 *g* 4 °C for 3–4 min to precipitate the cells, and the supernatant was discarded. Add 1 mL of JC-1 staining buffer to resuspend the cells, centrifuge at 600 *g* for 3–4 min at 4 °C, precipitate the cells and discard the supernatant. Finally, after resuspension with appropriate amount of JC-1 staining buffer, the red-green fluorescence ratio was detected by flow cytometry (BD, NJ, USA).

### LC/MS-MS mass spectrometry-based proteomics

The sample was sonicated three times on ice using a high-intensity ultrasonic processor (Scientz, Zhejiang, China) in lysis buffer (8 mol/L urea, 1% protease inhibitor cocktail). The remaining debris was removed by centrifugation at 12 000 *g* at 4 °C for 10 min. Finally, the supernatant was collected and the protein concentration was determined with BCA kit according to the manufacturer’s instructions.

For digestion, the protein solution was reduced with 5 mmol/L dithiothreitol for 30 min at 56 °C and alkylated with 11 mmol/L iodoacetamide for 15 min at room temperature in darkness. The protein sample was then diluted by adding 100 mmol/L TEAB to urea concentration less than 2 mol/L. Finally, trypsin was added at 1:50 trypsin-to-protein mass ratio for the first digestion overnight and 1:100 trypsin-to-protein mass ratio for a second 4 h-digestion. Finally, the peptides were desalted by C18 SPE column.

The tryptic peptides were dissolved in solvent A (0.1% formic acid, 2% acetonitrile/in water), directly loaded onto a homemade reversed-phase analytical column (25-cm length, 75/100 μm i.d.). Peptides were separated with a gradient from 6% to 24% solvent B (0.1% formic acid in acetonitrile) over 70 min, 24%–35% in 14 min and climbing to 80% in 3 min then holding at 80% for the last 3 min, all at a constant flow rate of 450 nL/min on a nanoElute UHPLC system (Bruker Co., MA, USA).

The peptides were subjected to capillary source followed by the timsTOF Pro (Bruker Co., MA, USA) mass spectrometry. The electrospray voltage applied was 1.60 kV. Precursors and fragments were analyzed at the TOF detector, with a MS/MS scan range from 100 to 1 700 m/z. The timsTOF Pro was operated in parallel accumulation serial fragmentation (PASEF) mode. Precursors with charge states 0–5 were selected for fragmentation, and 10 PASEF-MS/MS scans were acquired per cycle. The dynamic exclusion was set to 30 s.

The resulting MS/MS data were processed using MaxQuant search engine (v.1.6.15.0). Tandem mass spectra were searched against the human SwissProt database (20422 entries) concatenated with reverse decoy database. Trypsin/P was specified as cleavage enzyme allowing up to two missing cleavages. The mass tolerance for precursor ions was set as 20 × 10^−6^ in the first search and 5 × 10^–6^ in the main search, and the mass tolerance for fragment ions was set as 0.02 Da. Carbamidomethyl on Cys was specified as a fixed modification, and acetylation on protein N-terminal and oxidation on Met were specified as variable modifications. FDR was adjusted to <1%.

### Molecular docking

*HDAC3*–*Parkin* complex predictions were performed by Novopro, Guangdong, China. Briefly, 3D modeling of *HDAC3* and *Parkin* was conducted in I-TASSER software. *HDAC3* and *Parkin* docking was performed in BIOVIA Discovery Studio Visualizer software using ZDOCK algorithms. To gain more successive protein complex prediction, we reranked poses with the ZRANK scoring program: poses with a high density, high ZDOCK score and low ZRANK score were selected. The geometry of the selected docking solution was optimized using an energy minimization protocol and the Biovia Smart Minimizer algorithm. For the selected minimized solution, binding interface residues were identified and the types of interaction (for example, hydrogen bonds and electrostatic and hydrophobic interactions) were determined.

### Statistical analysis

All experiments were performed at least three times. All statistical analyses were performed using SPSS Statistics for Windows, Version 28.0 (IBM Corp., NY, USA), and graphs were generated with GraphPad Prism 8.0. Except where indicated, data are displayed as means, with uncertainty expressed as 95% confidence intervals (mean ± 95% CI). For unpaired experiments, two-tailed Student *t* test, linear regression analysis, or two-way ANOVA was performed. For paired experiments, two-tailed paired *t* test or linear mixed effect models were utilized. We checked the features of the regression model by comparing the residuals vs. fitted values (i.e., the residuals had to be normally distributed around zero) and independence between observations. No correction was applied for multiple comparison because outcomes were determined a priori and were highly correlated. No statistical analyses included confounders (e.g., body mass in each animal) due to the small sample size. We conducted a complete case analysis in the case of missing data. In all experiments, *P* values < 0.05 were considered statistically significant. Throughout this text, “*n*” represents the number of independent observations of knees or cells from different animals. Specific data representation details and statistical procedures are also indicated in the figure legends.

### Supplementary information

Supplemental material

## Data Availability

Data are available in a public, open-access repository. The mass spectrometry proteomics data have been deposited to the ProteomeXchange Consortium via the PRIDE^53^ partner repository with the dataset identifier PXD046993. All data generated or analyzed during this study are included in this submitted article. and its additional files.

## References

[1] Martel-Pelletier, J. et al. Osteoarthritis. *Nat. Rev. Dis. Prim.* **2**, 16072 (2016). 10.1038/nrdp.2016.72 27734845

[2] Sharma L (2021). Osteoarthritis of the knee. N. Engl. J. Med..

[3] Glyn-Jones S (2015). Osteoarthritis. Lancet.

[4] Zhang H (2023). Maintaining hypoxia environment of subchondral bone alleviates osteoarthritis progression. Sci. Adv..

[5] Loeser RF, Collins JA, Diekman BO (2016). Ageing and the pathogenesis of osteoarthritis. Nat. Rev. Rheumatol..

[6] De Belly H, Paluch EK, Chalut KJ (2022). Interplay between mechanics and signalling in regulating cell fate. Nat. Rev. Mol. Cell Biol..

[7] Iijima H (2023). Age-related matrix stiffening epigenetically regulates α-Klotho expression and compromises chondrocyte integrity. Nat. Commun..

[8] Armiento AR, Alini M, Stoddart MJ (2019). Articular fibrocartilage—why does hyaline cartilage fail to repair?. Adv. Drug Deliv. Rev..

[9] Jiang W (2021). Mechanisms linking mitochondrial mechanotransduction and chondrocyte biology in the pathogenesis of osteoarthritis. Ageing Res. Rev..

[10] Richard D (2020). Evolutionary selection and constraint on human knee chondrocyte regulation impacts osteoarthritis risk. Cell.

[11] Zhang H (2022). Mechanical overloading promotes chondrocyte senescence and osteoarthritis development through downregulating FBXW7. Ann. Rheum. Dis..

[12] Wang S (2022). Mechanical overloading induces GPX4-regulated chondrocyte ferroptosis in osteoarthritis via Piezo1 channel-facilitated calcium influx. J. Adv. Res..

[13] Han S (2022). Osteoarthritis year in review 2022: biology. Osteoarthr. Cartil..

[14] Peng Z (2021). The regulation of cartilage extracellular matrix homeostasis in joint cartilage degeneration and regeneration. Biomaterials.

[15] Kim JH (2015). Matrix cross-linking-mediated mechanotransduction promotes posttraumatic osteoarthritis. Proc. Natl. Acad. Sci. USA.

[16] Stolz M (2009). Early detection of aging cartilage and osteoarthritis in mice and patient samples using atomic force microscopy. Nat. Nanotechnol..

[17] Wu B (2023). Stiff matrix induces exosome secretion to promote tumour growth. Nat. Cell Biol..

[18] Patwardhan S, Mahadik P, Shetty O, Sen S (2021). ECM stiffness-tuned exosomes drive breast cancer motility through thrombospondin-1. Biomaterials.

[19] Xu M (2017). Transplanted senescent cells induce an osteoarthritis-like condition in mice. J. Gerontol. Ser. A Biol. Sci. Med. Sci..

[20] Xie J (2021). Cellular senescence in knee osteoarthritis: molecular mechanisms and therapeutic implications. Ageing Res. Rev..

[21] Jeon OH (2017). Local clearance of senescent cells attenuates the development of post-traumatic osteoarthritis and creates a pro-regenerative environment. Nat. Med..

[22] Hayashi D (2014). Pre-radiographic osteoarthritic changes are highly prevalent in the medial patella and medial posterior femur in older persons: Framingham OA study. Osteoarthr. Cartil..

[23] Engeland K (2022). Cell cycle regulation: p53-p21-RB signaling. Cell Death Differ..

[24] Baker DJ (2011). Clearance of p16Ink4a-positive senescent cells delays ageing-associated disorders. Nature.

[25] Meng F (2018). MicroRNA-193b-3p regulates chondrogenesis and chondrocyte metabolism by targeting HDAC3. Theranostics.

[26] Esteban-Martínez L, Boya P (2018). BNIP3L/NIX-dependent mitophagy regulates cell differentiation via metabolic reprogramming. Autophagy.

[27] Peña-Blanco A (2020). Drp1 modulates mitochondrial stress responses to mitotic arrest. Cell Death Differ..

[28] Shaltouki A, Hsieh CH, Kim MJ, Wang X (2018). Alpha-synuclein delays mitophagy and targeting Miro rescues neuron loss in Parkinson’s models. Acta Neuropathol..

[29] Emmett MJ, Lazar MA (2019). Integrative regulation of physiology by histone deacetylase 3. Nat. Rev. Mol. Cell Biol..

[30] Narita T, Weinert BT, Choudhary C (2019). Functions and mechanisms of non-histone protein acetylation. Nat. Rev. Mol. Cell Biol..

[31] Sun X (2022). Histone deacetylase inhibitors inhibit cervical cancer growth through Parkin acetylation-mediated mitophagy. Acta Pharm. Sin. B.

[32] Yi B, Xu Q, Liu W (2022). An overview of substrate stiffness guided cellular response and its applications in tissue regeneration. Bioact. Mater..

[33] Janmey PA, Fletcher DA, Reinhart-King CA (2020). Stiffness sensing by cells. Physiol. Rev..

[34] Vining KH, Mooney DJ (2017). Mechanical forces direct stem cell behaviour in development and regeneration. Nat. Rev. Mol. cell Biol..

[35] Lyu C (2023). Advanced glycation end-products as mediators of the aberrant crosslinking of extracellular matrix in scarred liver tissue. Nat. Biomed. Eng..

[36] Zeng L (2006). HDAC3 is crucial in shear- and VEGF-induced stem cell differentiation toward endothelial cells. J. Cell Biol..

[37] Wallace DC (2013). A mitochondrial bioenergetic etiology of disease. J. Clin. Investig..

[38] Forbes JM, Thorburn DR (2018). Mitochondrial dysfunction in diabetic kidney disease. Nat. Rev. Nephrol..

[39] Sorrentino V, Menzies KJ, Auwerx J (2018). Repairing mitochondrial dysfunction in disease. Annu. Rev. Pharmacol. Toxicol..

[40] Palikaras K, Lionaki E, Tavernarakis N (2018). Mechanisms of mitophagy in cellular homeostasis, physiology and pathology. Nat. Cell Biol..

[41] Liu L (2023). The physiological metabolite α-ketoglutarate ameliorates osteoarthritis by regulating mitophagy and oxidative stress. Redox Biol..

[42] Jin Z (2022). Curcumin exerts chondroprotective effects against osteoarthritis by promoting AMPK/PINK1/Parkin-mediated mitophagy. Biomed. Pharmacother..

[43] Ansari MY, Khan NM, Ahmad I, Haqqi TM (2018). Parkin clearance of dysfunctional mitochondria regulates ROS levels and increases survival of human chondrocytes. Osteoarthr. Cartil..

[44] Wang C (2019). Protective effects of metformin against osteoarthritis through upregulation of SIRT3-mediated PINK1/Parkin-dependent mitophagy in primary chondrocytes. Biosci. Trends.

[45] Shin, H. J. et al. Pink1-mediated chondrocytic mitophagy contributes to cartilage degeneration in osteoarthritis. *J. Clin. Med.* **8**, 1849 (2019). 10.3390/jcm8111849 PMC6912334 31684073

[46] Kuang Z (2019). The intestinal microbiota programs diurnal rhythms in host metabolism through histone deacetylase 3. Science.

[47] Wang Z (2020). SETD5-coordinated chromatin reprogramming regulates adaptive resistance to targeted pancreatic cancer therapy. Cancer Cell.

[48] Petrucelli L (2002). Parkin protects against the toxicity associated with mutant alpha-synuclein: proteasome dysfunction selectively affects catecholaminergic neurons. Neuron.

[49] Wang R (2023). PINK1, Keap1, and Rtnl1 regulate selective clearance of endoplasmic reticulum during development. Cell.

[50] Tamaddon M (2020). Osteochondral scaffolds for early treatment of cartilage defects in osteoarthritic joints: from bench to clinic. Biomater. Transl..

[51] Donate R (2022). Translation through collaboration: practice applied in BAMOS project in in vivo testing of innovative osteochondral scaffolds. Biomater. Transl..

[52] Wang Y, Chen Y, Wei Y (2022). Osteoarthritis animal models for biomaterial-assisted osteochondral regeneration. Biomater. Transl..

[53] Perez-Riverol Y (2022). The PRIDE database resources in 2022: a hub for mass spectrometry-based proteomics evidences. Nucleic Acids Res..

