# Supplementary material for: Matrix stiffening promotes chondrocyte senescence and the osteoarthritis development through downregulating HDAC3
Source: Bone Res. 2024 May 24;12:32. doi: 10.1038/s41413-024-00333-9 (PMC11126418; doi:10.1038/s41413-024-00333-9)
Supplement: Supplementary file 1 — Supplemental material [file 41413_2024_333_MOESM1_ESM.docx]

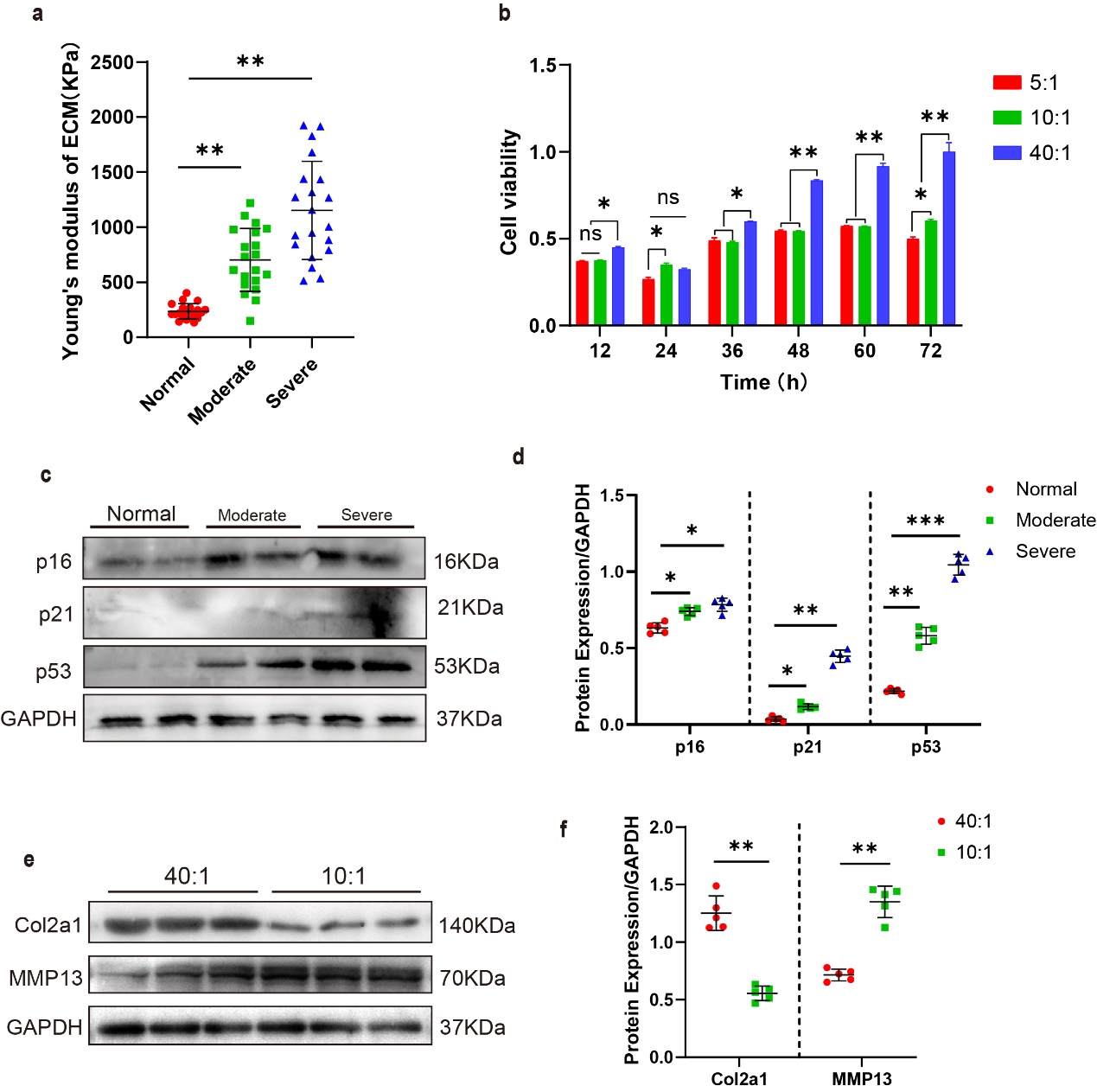


Supplementary Figure 1

ECM stiffening induced chondrocyte senescence in vitro and in mice. (A) Measurement of matrix stiffness (Young’s modulus) for normal and OA patients in moderately damaged and severely damaged using atomic force microscopy (AFM). n=20 per group. (B) Chondrocytes Viability Assessed by CCK-8 at 12, 24, 36, 48, 60, and 72 Hours After cultivated on PDMS substrates with Different Stiffness (40:1,10:1, 5:1). (C, D) Western Blotting analysis of *p16*^INK4a^, *p21* and *p53* expression in articular cartilage from normal and OA patients in moderately damaged and severely damaged. n=5 per group. (E, F) Western Blotting analysis of *Col2a1* and *MMP13* expression in mouse primary chondrocytes cultivated on 40:1,10:1, 5:1PDMS for 48 hours. n=5 per group.

*P<0.05, **P<0.01, ***p<0.001. NS, not significant.


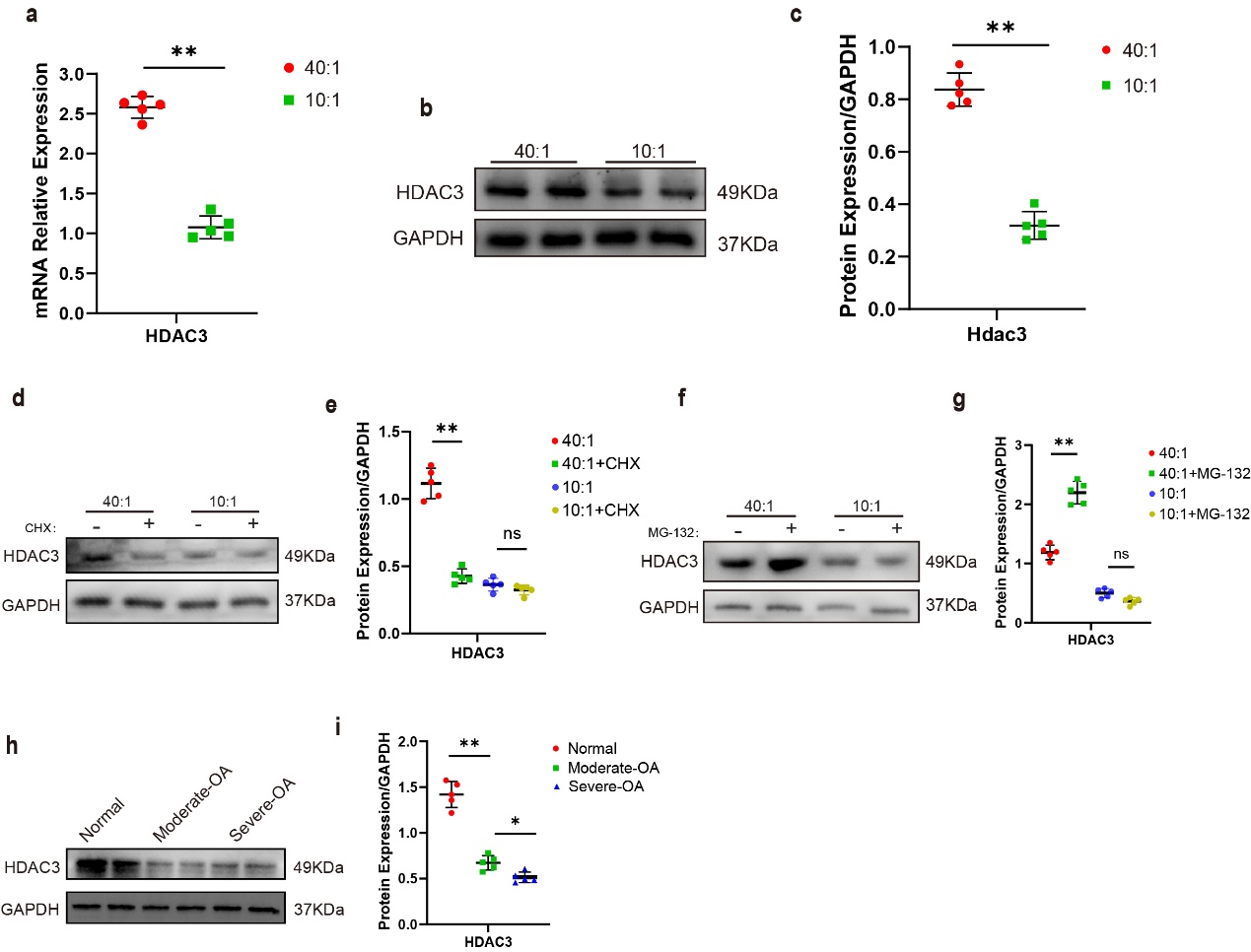


Supplementary Figure 2

Chondrocyte *HDAC3* is reduced by ECM stiffening and is decreased in the articular cartilage of OA patients. (A) Quantitative PCR analysis of *HDAC3* in the chondrocytes from human cultivated on Physiologic and Pathologic stiffness of PDMS substrates. n=5 per group. (B, C) Western Blotting analysis of *HDAC3* expression in chondrocytes from human cultivated on Physiologic and Pathologic stiffness of PDMS substrates. n=5 per group. (D, E) Western Blotting analysis of *HDAC3* expression in primary chondrocytes treated with CHX or not cultivated on 40:1 and 10:1 PDMS substrates. n=5 per group. (F, G) Western Blotting analysis of *HDAC3* expression in primary chondrocytes treated with MG-132 or not cultivated on 40:1 and 10:1 PDMS substrates. n=5 per group. (H, I) Western Blotting analysis of *HDAC3* expression in chondrocytes from normal and OA patients in moderately damaged and severely damaged. n=5 per group.

*P<0.05, **P<0.01. NS, not significant; 40:1, 10:1, 40:1, 10:1 polydimethylsiloxane (PDMS) substrates. OA, osteoarthritis; CHX, Cycloheximide.


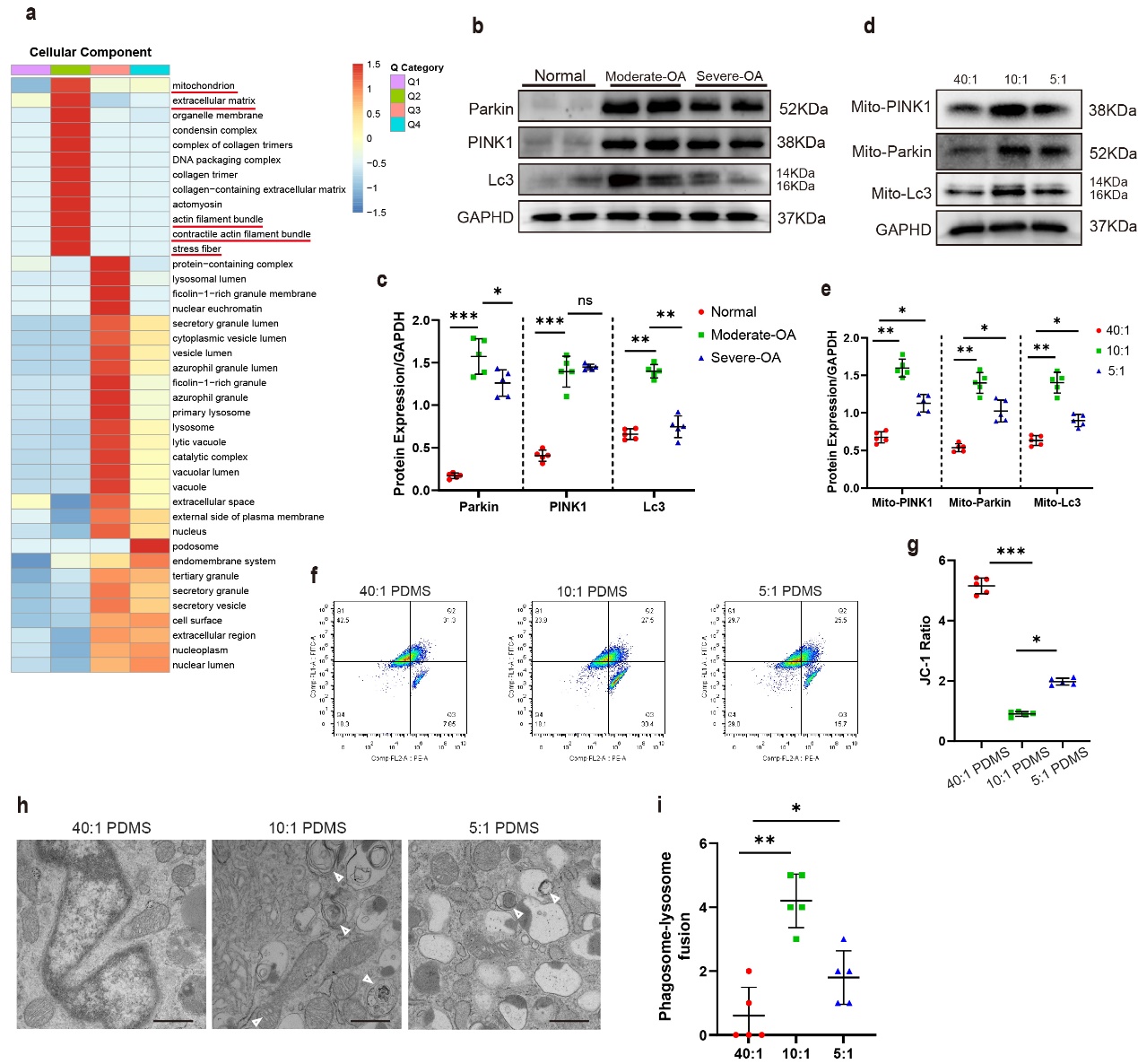


Supplementary Figure 3

ECM stiffening activates *PINK1*/*Parkin* mediated mitophagy. (A) Creation of a heatmap using hierarchical clustering to group related functions from different Q groups within cellular component, Based on Fisher's Exact Test P-values obtained from enrichment analysis. The horizontal axis represents different Q groups, while the vertical axis describes the cellular component associated with enriched differentially expressed proteins. (B, C) Western Blotting analysis of *PINK1*, *Parkin* and *Lc3* expression in articular cartilage from normal and OA patients in moderately damaged and severely damaged. n=5 per group. (D, E) Western Blotting analysis of *PINK1*, *Parkin* and *Lc3* expression in mitochondria of primary chondrocytes cultivated on 40:1, 10:1 and 5:1 PDMS substrates. n=5 per group. (F, G) Representative images and quantification of mitochondrial membrane potential by flow cytometry in chondrocytes cultivated on 40:1, 10:1 and 5:1 PDMS substrates. n=5 per group. (H) Representative images of morphology and number of chondrocyte mitochondria observed by transmission electron microscopy. Scale bars:1 µm. (I) Quantification of phagosome-lysosome fusion in chondrocytes based on results in (H). n=5 per group.

*P<0.05, **P<0.01, ***p<0.001. NS, not significant; 40:1, 10:1, 5:1, 40:1, 10:1, 5:1 polydimethylsiloxane (PDMS) substrates.


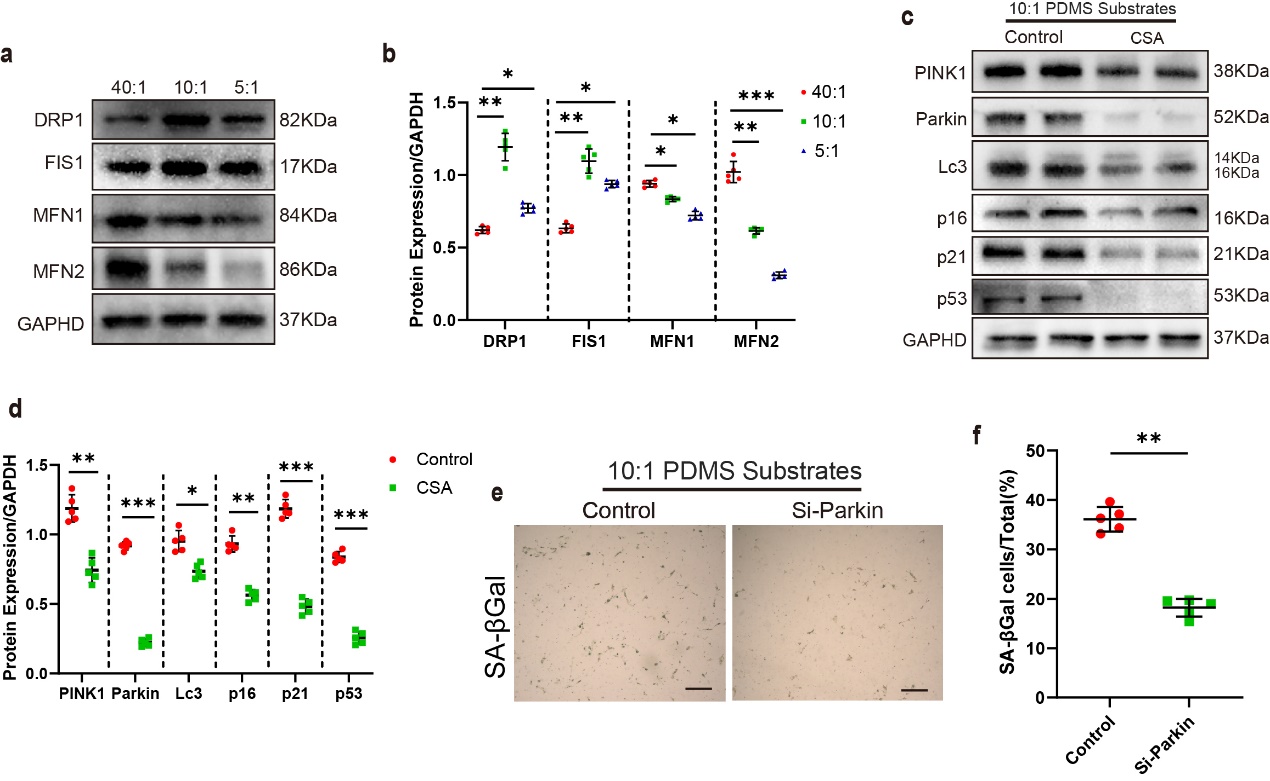


Supplementary Figure 4

The inhibition of *PINK1*/*Parkin* mediated mitophagy delays chondrocyte senescence and joint degeneration in mice. (A, B) Western Blotting analysis of *DRP1*, *FIS1*, *MFN1* and *MFN2* expression in primary chondrocytes cultivated on 40:1, 10:1 and 5:1 PDMS substrates. n=5 per group. (C, D) Western Blotting analysis of *PINK1*, *Parkin*, *Lc3,* *p16*^INK4a^, *p21* and *p53* expression in primary chondrocytes treated with CSA or not cultivated on 40:1 and 10:1 PDMS substrates. n=5 per group (E, F) Representative images and quantification of SA-βGal staining in primary chondrocytes which transfected si-NC or si-*Parkin* cultivated on 10:1 PDMS substrates. n=5 per group. Scale bar: 50 µm.

*P<0.05, **P<0.01, ***p<0.001.


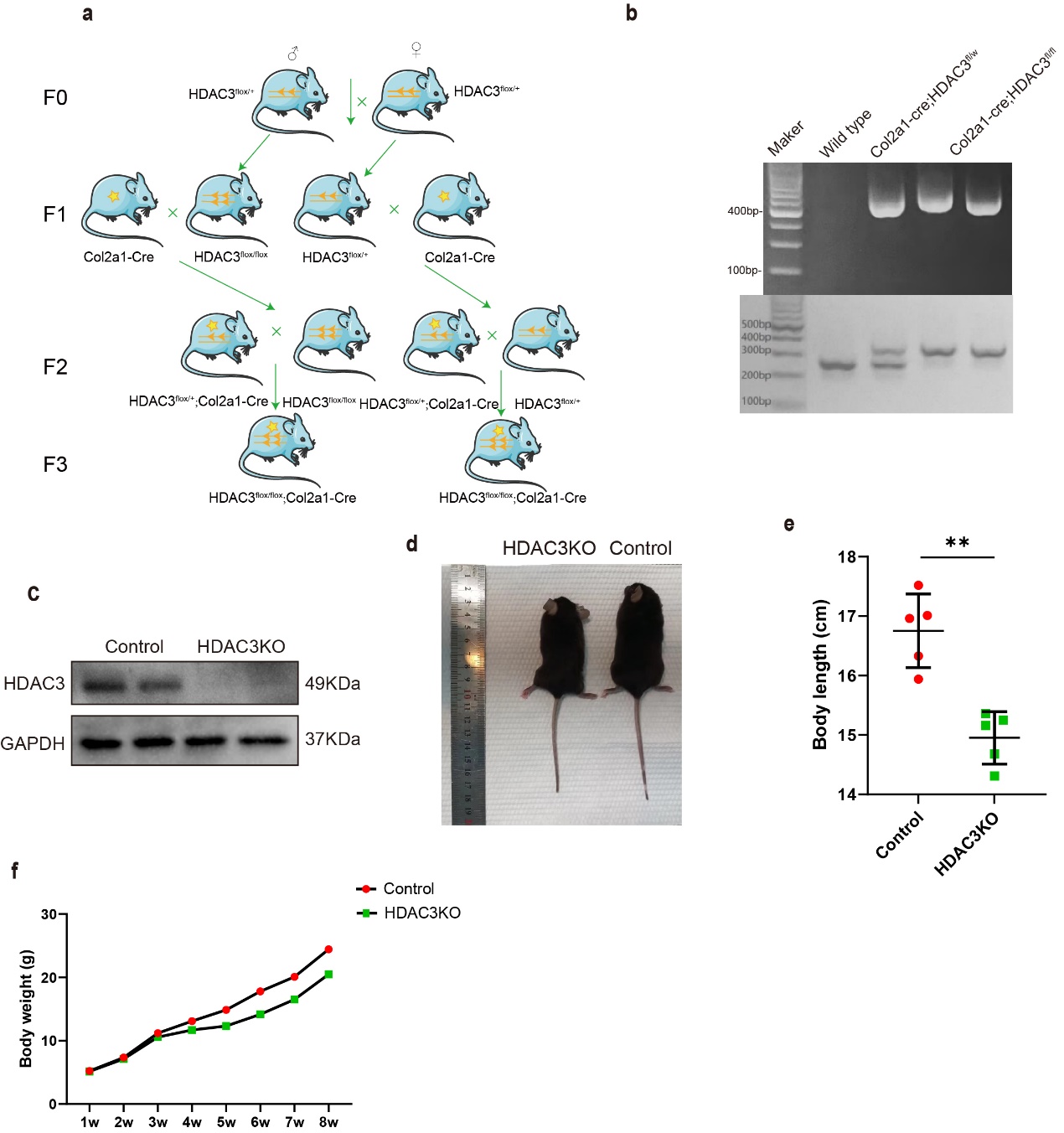


Supplementary Figure 5

Loss of *HDAC3* activates *PINK1*/*Parkin* signaling to promote chondrocyte senescence and OA progression. (A) *HDAC3* conditional knockout mouse construction process. (B) Genotyping the offspring after mating transgenic Cre and loxp mice. Original image of agarose gel electrophoresis. (C) Western blot of *HDAC3* in articular cartilage from *HDAC3*KO mice and controls. (D) General phenotypes of control and *HDAC3*KO mice at 8 weeks of age. (E) Quantification of the body length of control and *HDAC3*KO mice at 8 weeks of age. n = 5 per group. (F) Graph of control and *HDAC3*KO mice body weights from postnatal week 1 to week 8. n = 5 per group.


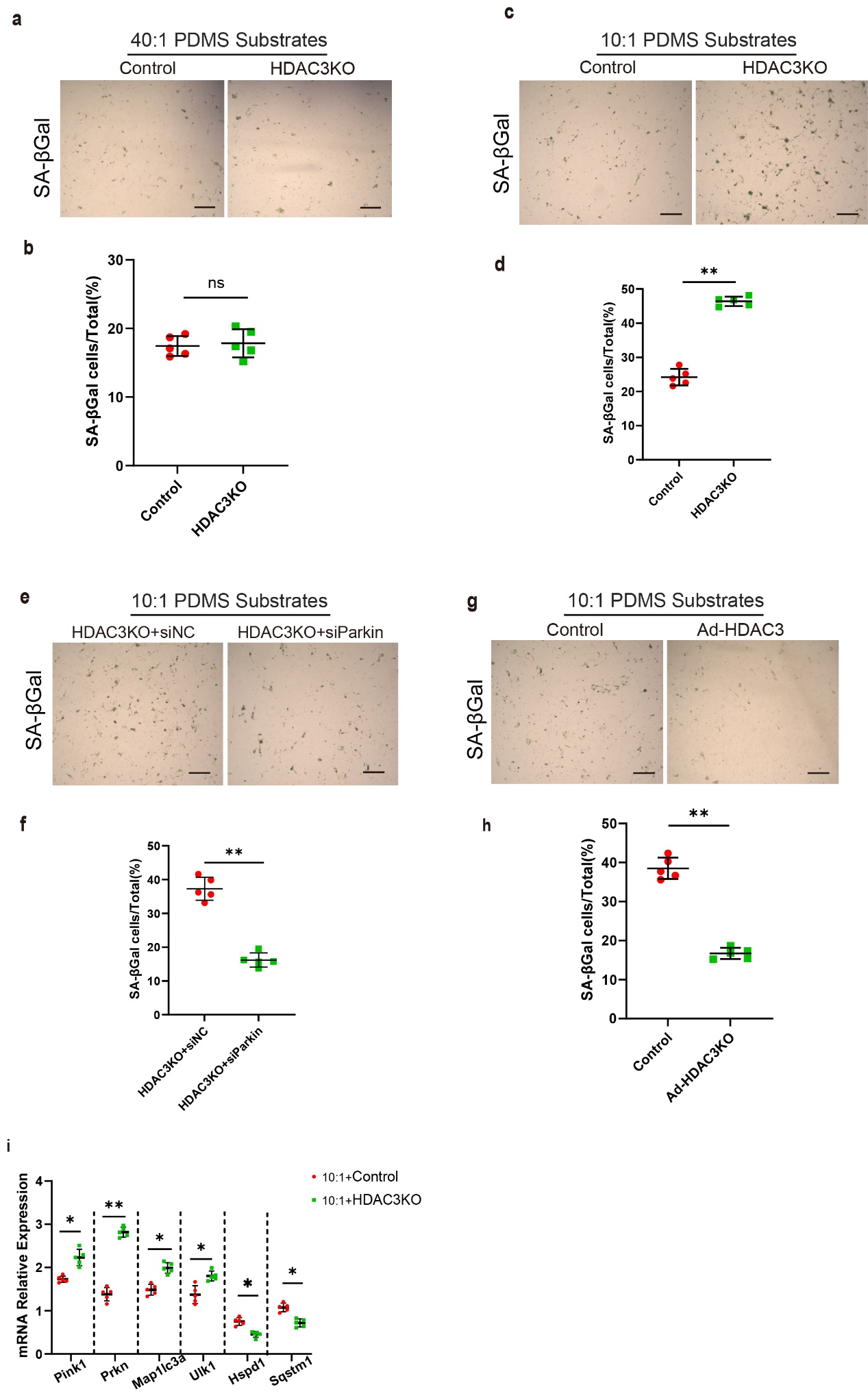


Supplementary Figure 6

(A, B) Representative images and quantification of SA-βGal staining in primary chondrocytes of control and *HDAC3*KO mice cultivated on 10:1 PDMS substrates. n=5 per group. Scale bar: 50 µm. (C, D) Representative images and quantification of SA-βGal staining in primary chondrocytes of control and *HDAC3*KO mice cultivated on 10:1 PDMS substrates. n=5 per group. Scale bar: 50 µm. (E, F) Representative images and quantification of SA-βGal staining in *HDAC3*KO and Control primary chondrocytes cultivated on 10:1 PDMS substrates for 48 hours. n=5 per group. Scale bar: 50 µm. (G, H) Representative images and quantification of SA-βGal staining in primary chondrocytes which transfected Ad-NC or Ad-*HDAC3 cult*ivated on 10:1 PDMS substrates. n=5 per group. Scale bar: 50 µm. (I) Quantitative PCR analysis of *PINK1*, *Prkn*, *Map1Lc3a*, *Ulk1*, *Hspd1*, *Sqstm1* in the primary chondrocytes from *HDAC3*KO mice and littermate Controls cultivated on 40:1 PDMS substrates. n=5 per group.

**P<0.01, NS, not significant;
